# Supplementary material for: Ranking microbial metabolomic and genomic links in the NPLinker framework using complementary scoring functions
Source: PLoS Comput Biol. 2021 May 4;17(5):e1008920. doi: 10.1371/journal.pcbi.1008920 (PMC8130963; doi:10.1371/journal.pcbi.1008920)
Supplement: S1 Text — (PDF) [file pcbi.1008920.s001.pdf]

Ranking microbial metabolomic and genomic links in the NPLinker framework using complementary scoring functions

Supplementary information

Grímur Hjörleifsson Eldjárn,
Andrew Ramsay,
Justin J. J. van der Hooft,
Katherine R. Duncan,
Sylvia Soldatou,
Juho Rousu,
Rónán Daly,
Joe Wandy,
and Simon Rogers

April 28, 2021

Appendix A Strain correlation score p-value

Consider the population of strains as a set N, with cardinality $\#N$, and consider the GCF G and molecular family M as subsets of N. A strain correlation scoring function σ is a function taking as arguments two subsets $G, M \subseteq N$, representing the GCF and the MF respectively, and assigning to the pair a real-valued score s.

The scoring function we use is the scoring function defined in [1], which is defined as

$$\begin{aligned}\sigma(M,G) =& \alpha\#(M\cap G) + \beta\#(N\setminus(M\cup G)) + \gamma\#(G\setminus(M\cap G)) + \delta\#(M\setminus(M\cap G)) && (1)\\ =& \alpha\#(M\cap G) + \beta(\#N - \#(M\cup G)) + \gamma(\#G - \#(M\cap G)) + \delta(\#M - \#(M\cap G)) && (2)\end{aligned}$$

where $\alpha = 10$, $\beta = -10$, $\gamma = 1$ and $\delta = 0$. The coefficients α, β, γ and δ correspond to the strain being in both the GCF and MF sets (α), neither of the sets (β), the GCF set but not the MF set (γ) and the MF set but not the GCF set (δ).

Given a GCF G and a MF M, and a scoring function σ, we want to be able to calculate the p-value for the potential link between G and M, i.e. the probability that $\sigma(M, G) > s$ under the assumption that G and M are independent. This means that for our purposes, the sizes of M and G, $\#M$ and $\#G$, are constants, as well as the total population size, $\#N$.

Assuming that M and G are independent, we have given only $\#M$ and $\#G$, and not $\#(M \cap G)$. Therefore, we have

$$p(\sigma(M,G) = s) = \sum_{o\in\mathbb{N}} p(\#(M\cap G) = o)p(\hat{\sigma}(o) = s) \tag{3}$$

We can therefore calculate the p-value as

$$\begin{aligned}p(\sigma(M,G) > s) &= \sum_{s'>s} p(\sigma(M,G) = s') && (4)\\ &= \sum_{s'>s} p(\sigma(M,G) = s' \mid \#M, \#G, \#N) && (5)\\ &= \sum_{s'>s}\sum_{o\in\mathbb{N}} p(\#(M\cap G) = o)p(\hat{\sigma}(o) = s') && (6)\end{aligned}$$

But since the score is determined completely by $\#M$, $\#G$, $\#(M \cap G)$ and $\#N$, the last term is always either 0 or 1. Furthermore,

$$p(\hat{\sigma}(\#(M\cap G)) = s') = 1 \tag{7}$$

precisely for those values of $\#(M \cap N)$ where

$$\hat{\sigma}(\#(M\cap G)) = s'.$$

Assuming as we are that M and G are independent, the first term, $p(\#(M \cap G) = o)$, can be considered, in terms of a hypergeometric distribution, as the probability of o "successes" in $\#M$ draws from a population of $\#N$, with a total of $\#G$ marked elements, i.e.

$$p(\#(M\cap G) = o) = p(o \mid \#M, \#G, \#N)$$

follows hypergeometric distribution.

The two sums can be taken together as

$$\sum_{o\mid\hat{\sigma}(o)>s} p(o \mid \#M, \#G, \#N)$$

where $o = \#(M \cap G)$. As we are assuming that M and G are independent, $p(o)$ follows hypergeometric distribution and can be calculated by standard means.

In practice, the calculations only need to be carried out for the values of o which are possible given $\#M$, $\#G$ and $\#N$, i.e. the lower bound for o is $max(0, (\#M + \#G) - \#N)$, and the upper bound is $min(\#M, \#G)$.

Appendix B The NPLinker Framework

NPLinker http://www.github.com/sdrogers/nplinker is intended to address the significant bottleneck that exists in the realization of the potential of genome-led metabolite discovery, namely the slow manual matching of predicted biosynthetic gene clusters (BGCs) with metabolites produced during bacterial culture; linking phenotype to genotype.

The NPLinker tool and its associated web application implement a new data-centric approach to alleviate this linking problem by searching for patterns of strain presence and absence between groups of similar spectra (molecular families; MF) and groups of similar BGCs (gene cluster families; GCF). Searching can be performed based on a number of available analysis methods that can be employed in isolation or combined as required.

NPLinker is implemented as a standard Python package, appropriate for use in scripting or for interactive environments such as a Jupyter notebook.

On top of this package, we have developed a Dockerised NPLinker web application which allows users to run it in a browser and avoid the process of installing and configuring a suitable local Python environment. Interactive HTML widgets (e.g. buttons, tables, sliders) provide a desktop-like interface while a backend Python process responds to the events triggered in the browser.

In order to use the web application, all that is required from the user is a suitable dataset. These may be drawn from the Paired omics Data Platform, from wholly local data, or a combination of the two. In the case of the Paired omics Data Platform the web application is capable of automated downloading and preprocessing, including running BiG-SCAPE if necessary. Output from BiG-SCAPE and other potentially lengthy analysis processes is cached locally to reduce the time required to launch the application after the initial run.

The user interface is shown in Fig A. From left to right the tables contain MFs, spectra, BGCs, and GCFs. The content is determined by an initial run of a standardised correlation scoring on the entire dataset with a user-configurable threshold. This is intended to remove the large number of original objects that are very unlikely to have any significant links. Subsequently when the user makes a selection of one or more objects from any table, all objects that are not linked to the selected object(s) are hidden. For example, selection of a MF will filter out all spectra not contained in that family. This in turn filters out all BGCs that do not share strains with those spectra. Finally, all GCFs that do not contain the BGCs are removed. The filtering operates similarly when other object types are selected as a starting point. This makes it possible for the user to rapidly look up and explore interesting objects in a dataset before running other scoring methods on the filtered results. At any point the user can also click a button to export a CSV file containing the current data displayed in any of the 4 tables for external analysis.

NPLinker contains all functionality described in the paper, as well as an additional scoring system named “Rosetta scoring”. This uses a hand-curated translation table between a small subset of GNPS library spectra, and their BGCs as stored in MiBIG. Putative links between a BGC and a spectrum in a data set can be highlighted where the spectrum shows similarity with a GNPS library spectrum in the Rosetta set and the BGC has homology to the corresponding MiBIG entry.

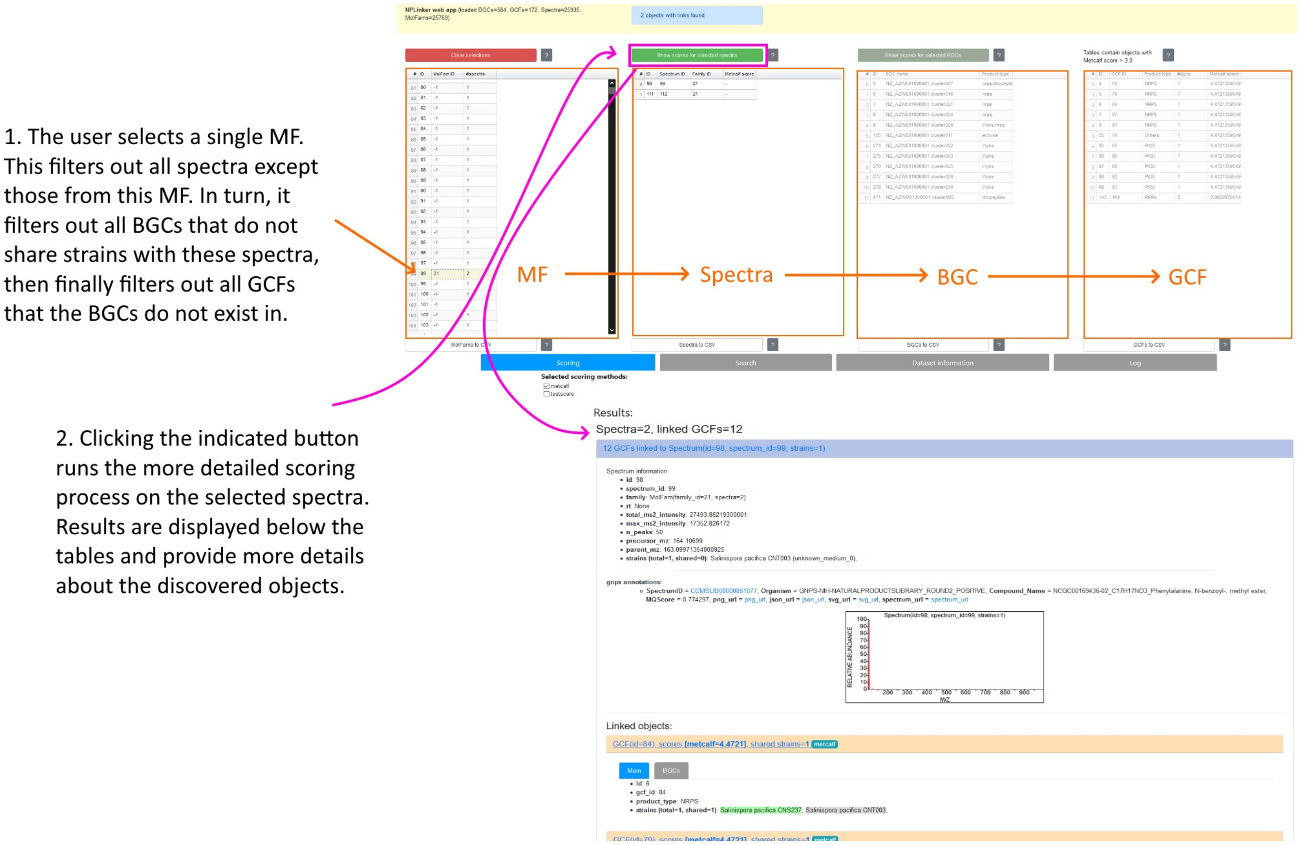

Figure A: Web application workflow. The user selects an MF from the table on the left, triggering filtering of spectra, BGCs, and GCFs in turn. The button above the spectra table is then clicked to generate results from the enabled scoring methods. The results are then displayed underneath the tables.

Appendix C NPLinker documentation

All Python source code for NPLinker is hosted on GitHub at https://github.com/sdrogers/nplinker.

An associated wiki at https://github.com/sdrogers/nplinker/wiki contains detailed instructions for installing and running the web application described above. The NPLinker web application Docker image is hosted on DockerHub.

API documentation for the NPLinker framework underlying the web application can be viewed at nplinker.readthedocs.io and is automatically updated when the source code changes. The GitHub repository also contains a heavily-commented Jupyter notebook which walks through the process of loading a dataset and using the NPLinker API to explore it and search for links.

Appendix D Matching predicted spectra to influential peaks

Appendix D.1 Rationale

To further validate the IOKR approach we investigated if it was possible, for high-scoring pairs of MS2 spectra and metabolites, to manually match relevant peaks in MS2 spectra to possible fragments of the metabolites. Full validation would require additional wet lab analysis, which is not possible with these publicly available datasets.

If a link is genuine, it ought to be possible to match MS2 peaks in the spectra to substructures of the relevant chemical structures. If we can, it ought to be the case that these fragment peaks are particularly important in the IOKR model. Below, we provide some examples to show that this is indeed the case.

Appendix D.2 Dataset

To illustrate this process, we took validated links in the Crusemann data set (see Section 2.8.2 and Table 4), as well as two high-scoring potential links chosen as their ranking had a strong contribution from the IOKR score.

Appendix D.3 Methodology

To match MS2 peaks to chemical substructures we made use of the MetFrag web interface [2]. For a given metabolite and spectrum, using the compound name search function within the NPAtlas database [3], we found the accurate mass for the metabolite. This was used as a search criterion on the neutral mass in the `NPAtlas_Aug2019` database in MetFrag [2], to ensure that the relevant metabolite was in the candidate set. Because we wanted to match measured peaks in an actual MS2 spectrum to the predicted peaks for a particular metabolite, ideally, the MetFrag candidate set should have one member. Where more than one result was returned, only the result where the candidate metabolite name matched the given metabolite was used, except in the case of griseochelin, which was considered equivalent to zincophorin as it has been by others in literature [4].

The relevant spectral data was extracted from the Metabolomics Spectrum Resolver [5] and the MetFrag in-silico fragmentation algorithm (with default settings) was used. Peaks that did match were then checked to see how their exclusion from the MS2 spectrum influenced the ranking of the metabolite, among the set of all metabolites, to that spectrum.

The images for the spectra were generated by the Metabolomics Spectrum Resolver [5] while the images for the metabolites were genreated by MetFrag [5], with the identified substructure highlighted in green.

Appendix D.4 Results

Example (validated link) For rosamicin, NPAtlas reported an accurate mass of 581.3564 which had a unique hit in the NPAtlas database in MetFrag. Of the 31 peaks in spectrum 93193, 12 were matched to the hypothetical spectrum, with a raw score of 67.1248. One of those, the peak at m/z 158.117004, matched the predicted peak at 158.11762 Da. Removing this peak changed the (0-based) rank of the metabolite against that spectrum from 16 to 163.

The predicted peak at 158.11762, highlighted in green in the image below, represents a biological subunit (aminosugar) of the metabolite.

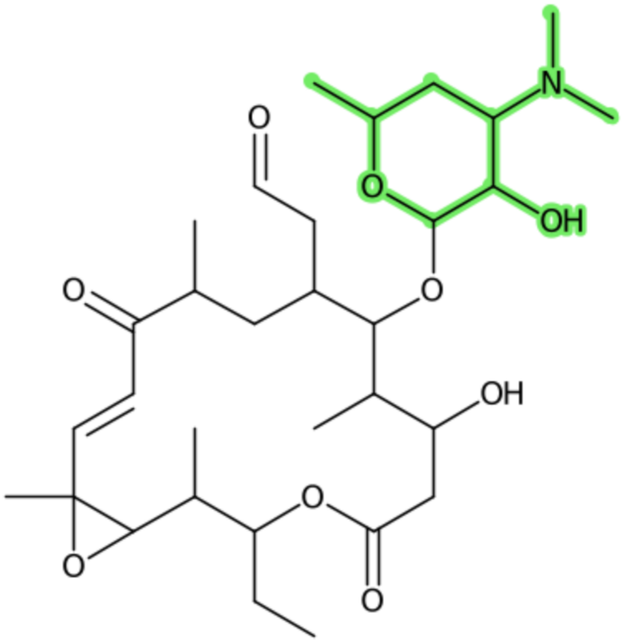

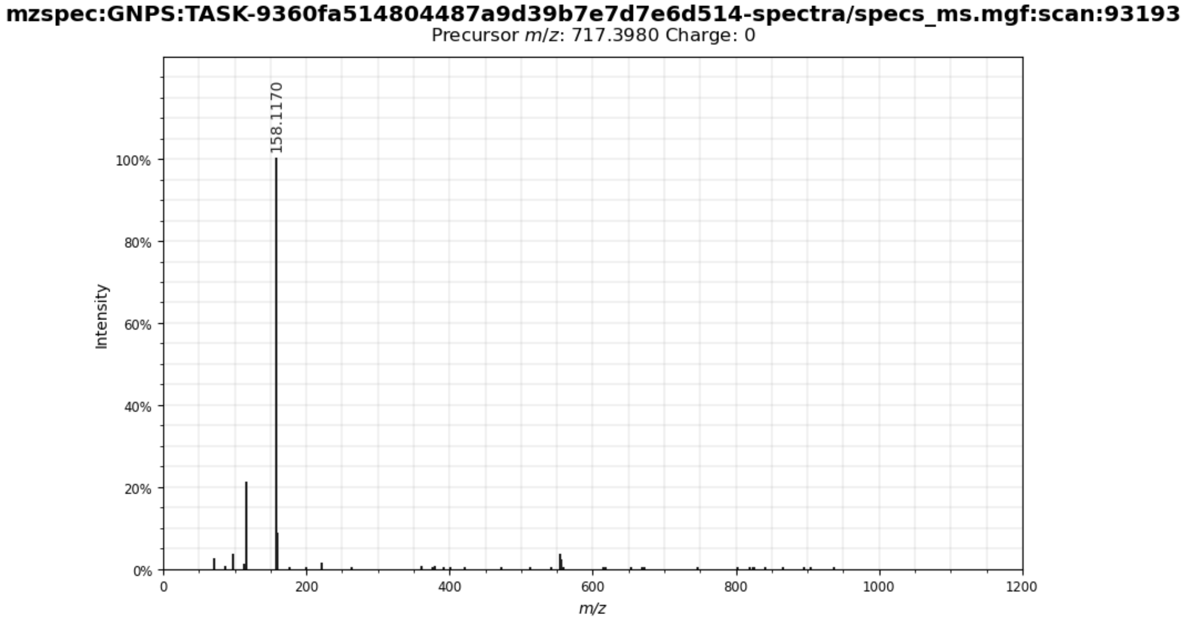

Example (potential link) For griseochelin, NPAtlas reported accurate mass of 568.4339, which yielded two candidates in the NPAtlas database in MetFrag. Curiously, when matching the spectra, only one result line showed up in MetFrag. This line was annotated as zincophorin, which is another name for griseochelin. Of the 18 peaks in spectrum 51165, 2 were matched to the hypothetical spectrum, with a raw score of 2.9841. One of those, the peak at m/z 95.086998, matched the predicted peak at 95.08558 Da, highlighted in green in the image below, and removing this peak changed the (0-based) rank of the metabolite against that spectrum from 0 to 3.

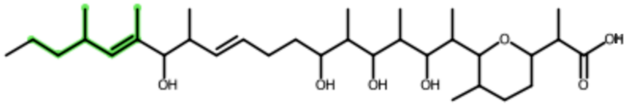

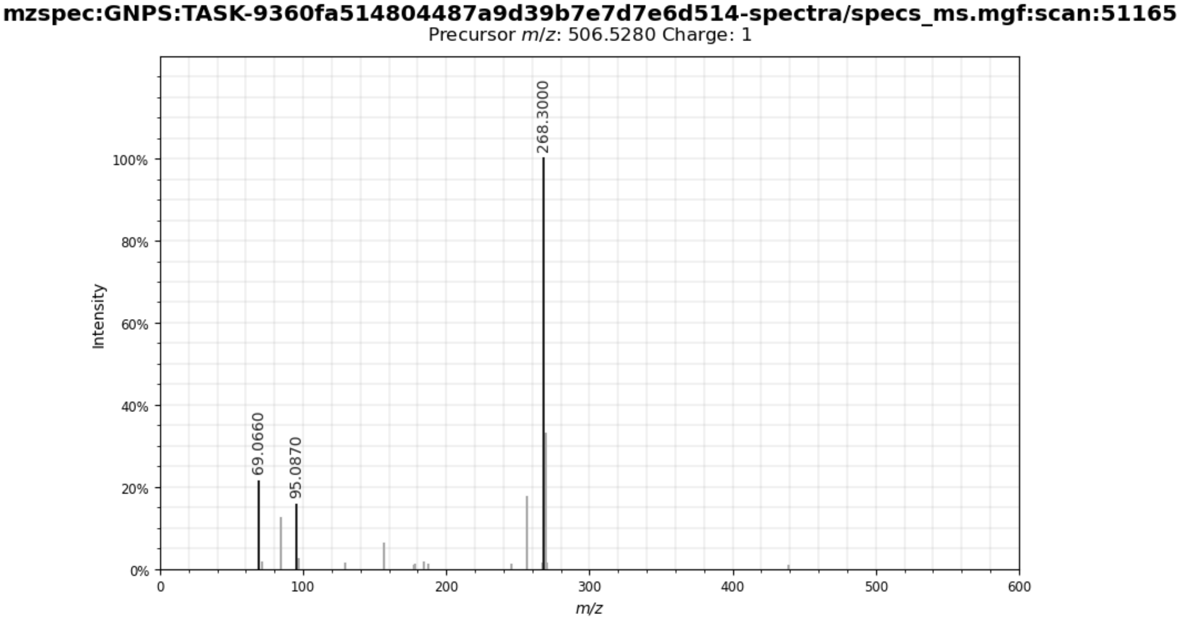

Example (validated link) For staurosporine, NPAtlas reported accurate mass of 466.2005, which yielded seven candidates from the NPAtlas database in MetFrag. Only one was annotated as staurosporine, and that was used for the analysis. Of the 22 peaks in spectrum 44982, 7 are matched to the hypothetical spectrum, with a raw score of 159.7769. One of those, the peak at m/z 56.049999, matched the predicted peak at 56.0495 Da, highlighted in green in the image below, and removing this peak changed the (0-based) rank of the metabolite against that spectrum from 1 to 67.

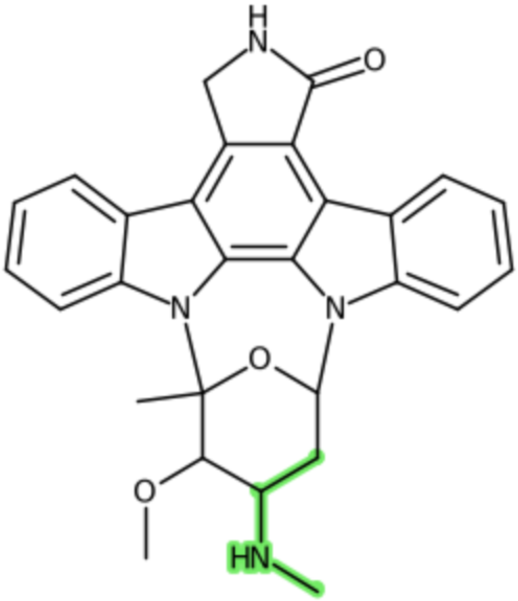

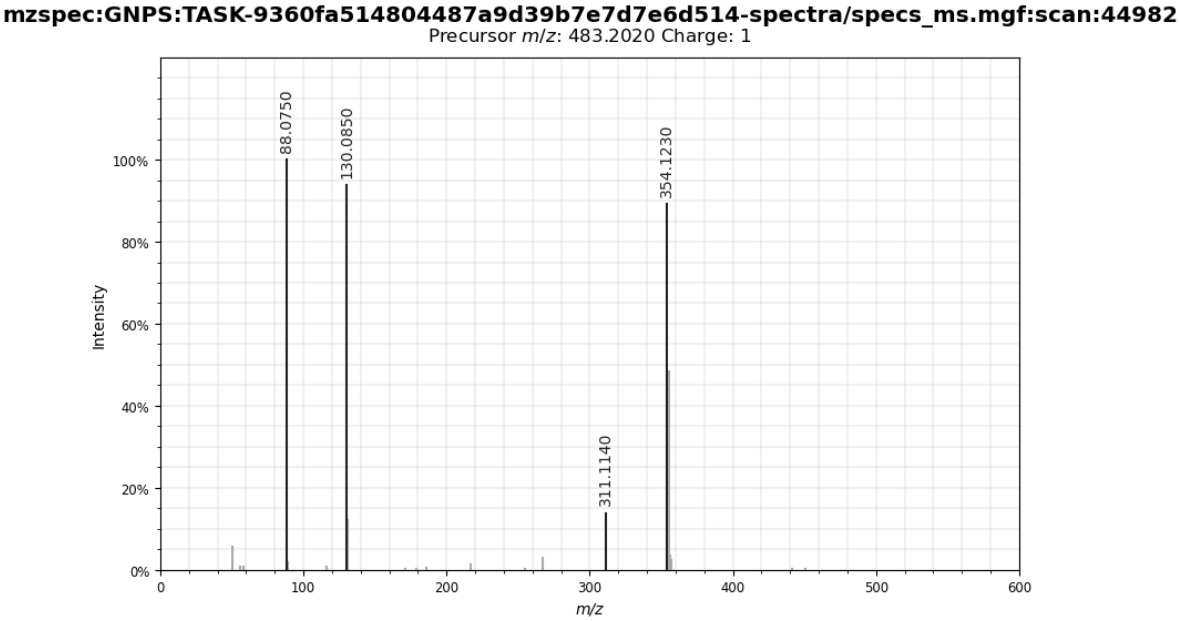

Table A Product types of validated links

Gross	size (nt)	type	no. genes
BGC0000632	16122	Terpene, Saccharide	13
BGC0001381	210303	Polyketide	102
BGC0001842	42814	NRP (other lipopeptide)	7
BGC0000463	43207	NRP (other lipopeptide)	4
BGC0001116	57999	NRP, Polyketide	18
BGC0000399	47020	NRP (cyclic depsipeptide)	17
BGC0000296	66086	NRP	28
BGC0001298	41001	Polyketide	9

Leão	size (nt)	type	no. genes
BGC0001165	87427	NRP, Polyketide (other)	14
BGC0000962	40156	NRP, Polyketide (other)	12
BGC0001000	41964	NRP (Other lipopeptide), Polyketide (other)	8
BGC0001001	69900	NRP, Polyketide (other)	26
BGC0001560	28792	NRP, Polyketide	12

Crüsemann	size (nt)	type	no. genes
BGC0000827	24300	Alkaloid	17
BGC0000333	47477	NRP	23
BGC0000940	7328	Other	6
BGC0000241	62231	Polyketide (Other), Saccharide (hybrid/tailoring)	58
BGC0001228	37781	NRP (Cyclic depsipeptide)	23
BGC0000137	91573	Polyketide	39
BGC0001830	64171	Polyketide	23

Table B Sizes of data sets

Sizes of the data sets, including how many BGCs could be structurally annotated for IOKR scoring using similarity to MIBiG entries. MIBiG entries are associated with BGCs by running antiSMASH in known cluster blast mode, and any homology detected by antiSMASH is considered valid.

	Crüsemann	Leão	Gross
Total BGCs	3316	147	131
BGCs with assigned structure	2242	57	83
Total spectra	6246	173	9593

Table C Comparison of score distributions

Comparison of mean scores for all links vs. validated links for the various data sets. The p-value is for the null hypothesis that the distributions have identical means.

Crüsemann	Mean score all	Mean score valid	p-value
Raw correlation	83.5144	14.6667	0.0001
Standardised correlation	-0.0060	3.6717	6.8302e-64
IOKR	0.0105	0.0364	1.7968e-9

Leão	Mean score all	Mean score valid	p-value
Raw correlation	-1.9843	12.625	0.0001
Standardised correlation	-0.0218	1.4962	1.1887e-05
IOKR	0.0014	0.0038	0.3922

Gross	Mean score all	Mean score valid	p-value
Raw correlation	-0.7386	0.6	0.7929
Standardised correlation	0.0092	1.6149	6.0056e-06
IOKR	0.02721	0.037020	0.5155

Table D Number of validated links in higher percentiles

Number of links (validated vs. total) for the different scoring functions and data sets. The first two columns describe the unfiltered data, while the others only count links scoring over 95th percentile on either or both scores.

	all		> 95% IOKR		> 95% correlation		> 95% both	
	verified	total	verified	total	verified	total	verified	total
Gross	5	501886	1	25095	2	35333	0	1537
Leão	8	9342	1	437	6	1560	1	77
Crüsemann	15	999362	6	49970	10	50224	4	2517
all	28	1510590	8	75502	18	87117	5	4131

	all		> 90% IOKR		> 90% correlation		> 90% both	
	verified	total	verified	total	verified	total	verified	total
Gross	5	501886	1	50189	4	52014	1	5313
Leão	8	9342	1	935	6	1560	1	147
Crüsemann	15	999362	6	99937	13	100494	5	10836
all	28	1510590	8	151061	23	154068	7	16296

References

[1] James R Doroghazi, Jessica C Albright, Anthony W Goering, Kou-San Ju, Robert R Haines, Konstantin A Tchalukov, David P Labeda, Neil L Kelleher, and William W Metcalf. A roadmap for natural product discovery based on large-scale genomics and metabolomics. *Nat. Chem. Biol.*, 10(11):963–968, November 2014.

[2] Christoph Ruttkies, Emma L. Schymanski, Sebastian Wolf, Juliane Hollender, and Steffen Neumann. Metfrag relaunched: incorporating strategies beyond in silico fragmentation. *Journal of Cheminformatics*, 8(1):3, Jan 2016.

[3] Jeffrey A. van Santen, Grégoire Jacob, Amrit Leen Singh, Victor Aniebok, Marcy J. Balunas, Derek Bunsko, Fausto Carnevale Neto, Laia Castaño-Espriu, Chen Chang, Trevor N. Clark, Jessica L. Cleary Little, David A. Delgadillo, Pieter C. Dorrestein, Katherine R. Duncan, Joseph M. Egan, Melissa M. Galey, F.P. Jake Haeckl, Alex Hua, Alison H. Hughes, Dasha Iskakova, Aswad Khadilkar, Jung-Ho Lee, Sanghoon Lee, Nicole LeGrow, Dennis Y. Liu, Jocelyn M. Macho, Catherine S. McCaughey, Marnix H. Medema, Ram P. Neupane, Timothy J. O'Donnell, Jasmine S. Paula, Laura M. Sanchez, Anam F. Shaikh, Sylvia Soldatou, Barbara R. Terlouw, Tuan Anh Tran, Mercia Valentine, Justin J. J. van der Hooft, Duy A. Vo, Mingxun Wang, Darryl Wilson, Katherine E. Zink, and Roger G. Linington. The natural products atlas: An open access knowledge base for microbial natural products discovery. *ACS Central Science*, 5(11):1824–1833, 2019.

[4] Elisabeth Walther, Sabrina Boldt, Hirokazu Kage, Tom Lauterbach, Karin Martin, Martin Roth, Christian Hertweck, Andreas Sauerbrei, Michaela Schmidtke, and Markus Nett. Zincophorin - biosynthesis in streptomyces griseus and antibiotic properties. *GMS infectious diseases*, 4:Doc08–Doc08, Nov 2016. 30671322[pmid].

[5] Wout Bittremieux, Christopher Chen, Pieter C. Dorrestein, Emma L. Schymanski, Tobias Schulze, Steffen Neumann, Rene Meier, Simon Rogers, and Mingxun Wang. Universal ms/ms visualization and retrieval with the metabolomics spectrum resolver web service. *bioRxiv*, 2020.
