# Supplementary material for: Ranking microbial metabolomic and genomic links in the NPLinker framework using complementary scoring functions
Source: PLoS Comput Biol. 2021 May 4;17(5):e1008920. doi: 10.1371/journal.pcbi.1008920 (PMC8130963; doi:10.1371/journal.pcbi.1008920)
Supplement: S1 Fig — Histograms showing the distribution of raw and standardised strain correlation scores for the microbial data sets, as well as positions of validated links within the distribution. (PDF) [file pcbi.1008920.s002.pdf]

Raw vs. standardised strain correlation scores

Comparison of raw and standardised strain correlation scores for the Crusemann, Leao and Gross data sets. Black lines represent the scores of validated links.

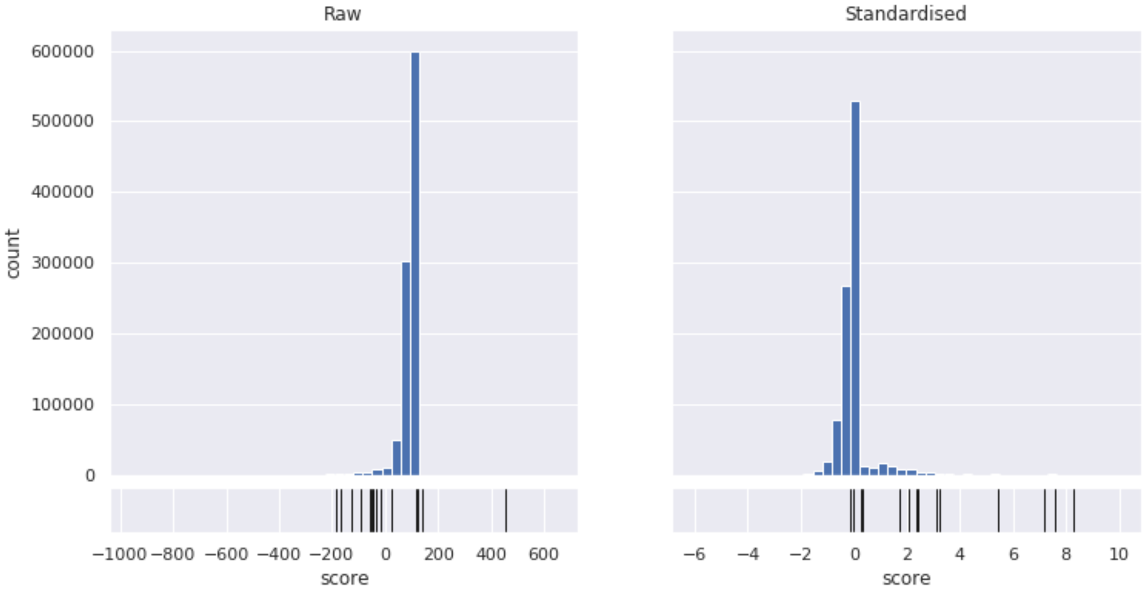

Crüsemann

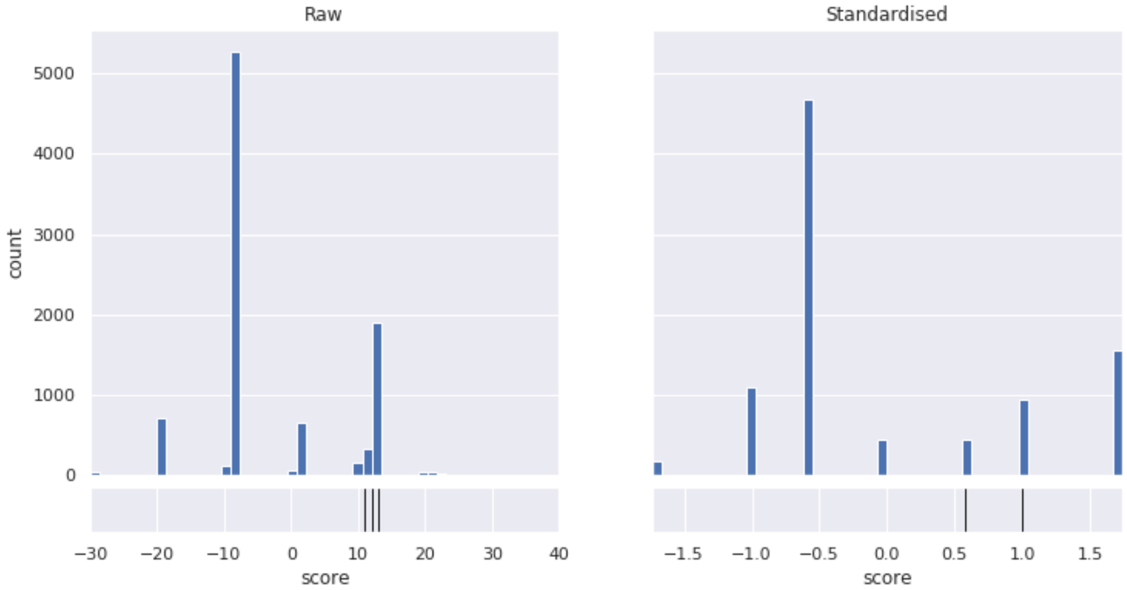

Leão

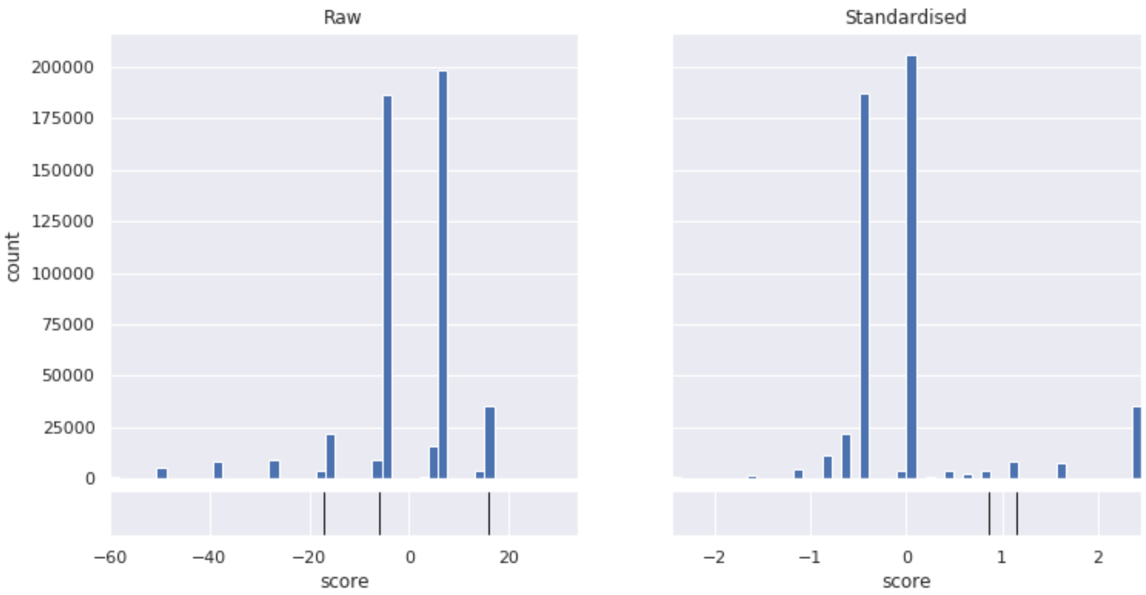
Raw
Standardised
count
200000
175000
150000
125000
100000
75000
50000
25000
0
−60
−40
−20
0
20
score
−2
−1
0
1
2
score

Gross
