# Supplementary material for: Ranking microbial metabolomic and genomic links in the NPLinker framework using complementary scoring functions
Source: PLoS Comput Biol. 2021 May 4;17(5):e1008920. doi: 10.1371/journal.pcbi.1008920 (PMC8130963; doi:10.1371/journal.pcbi.1008920)
Supplement: S2 Fig — Histograms of the distribution of IOKR scores for the microbial data sets, as well as positions of validated links within the distribution. (PDF) [file pcbi.1008920.s003.pdf]

IOKR score of validated links

Histograms of IOKR scores for the Crusemann, Leao and Gross data sets. Black lines represent the scores of validated links.

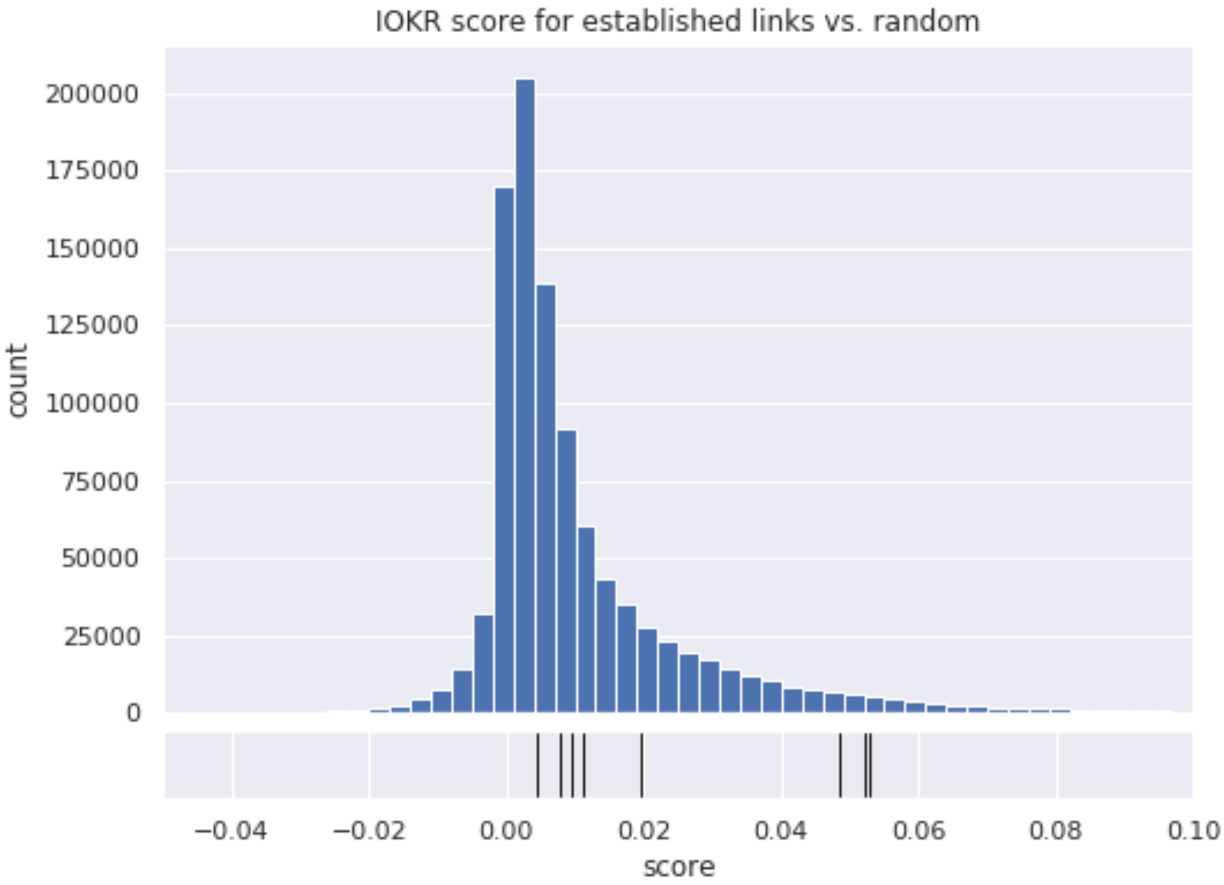

Crüsemann

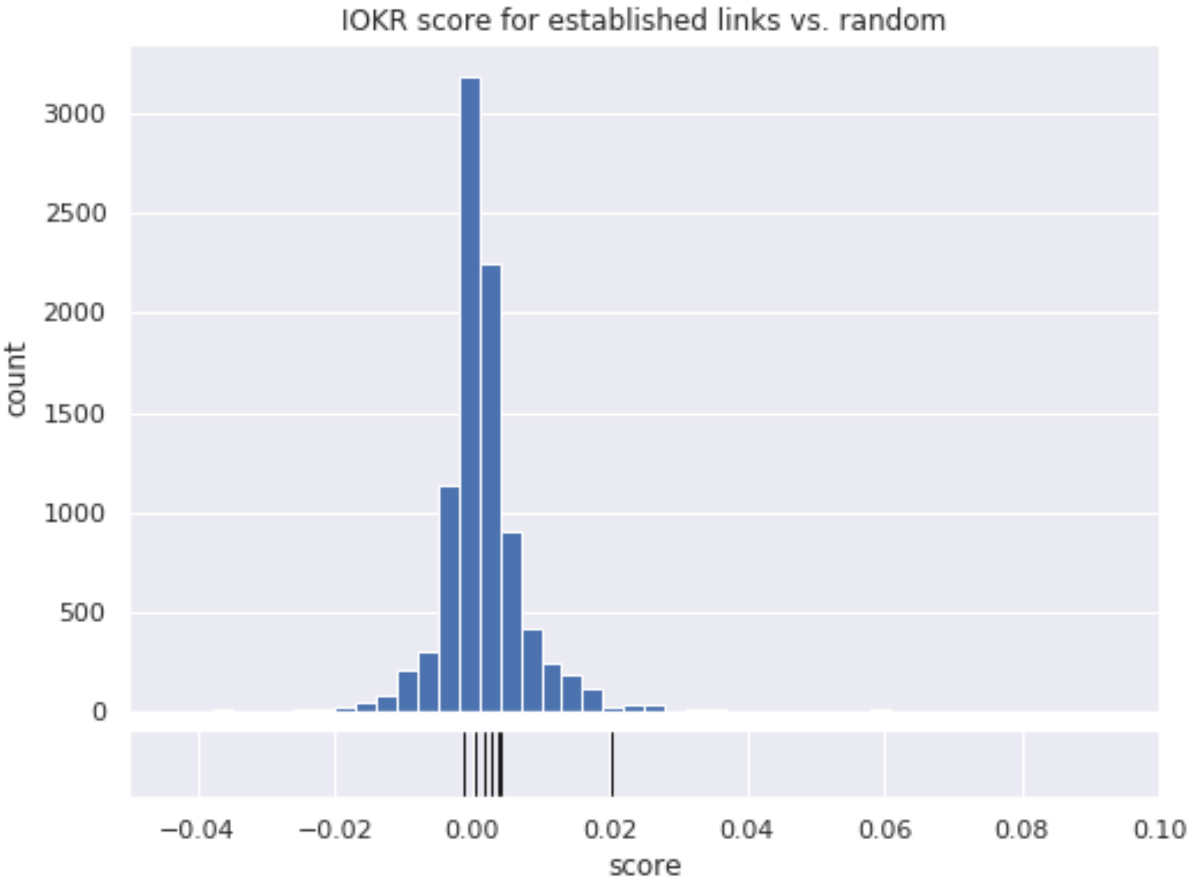
IOKR score for established links vs. random
count
3000
2500
2000
1500
1000
500
0
−0.04
−0.02
0.00
0.02
0.04
0.06
0.08
0.10
score

Leão

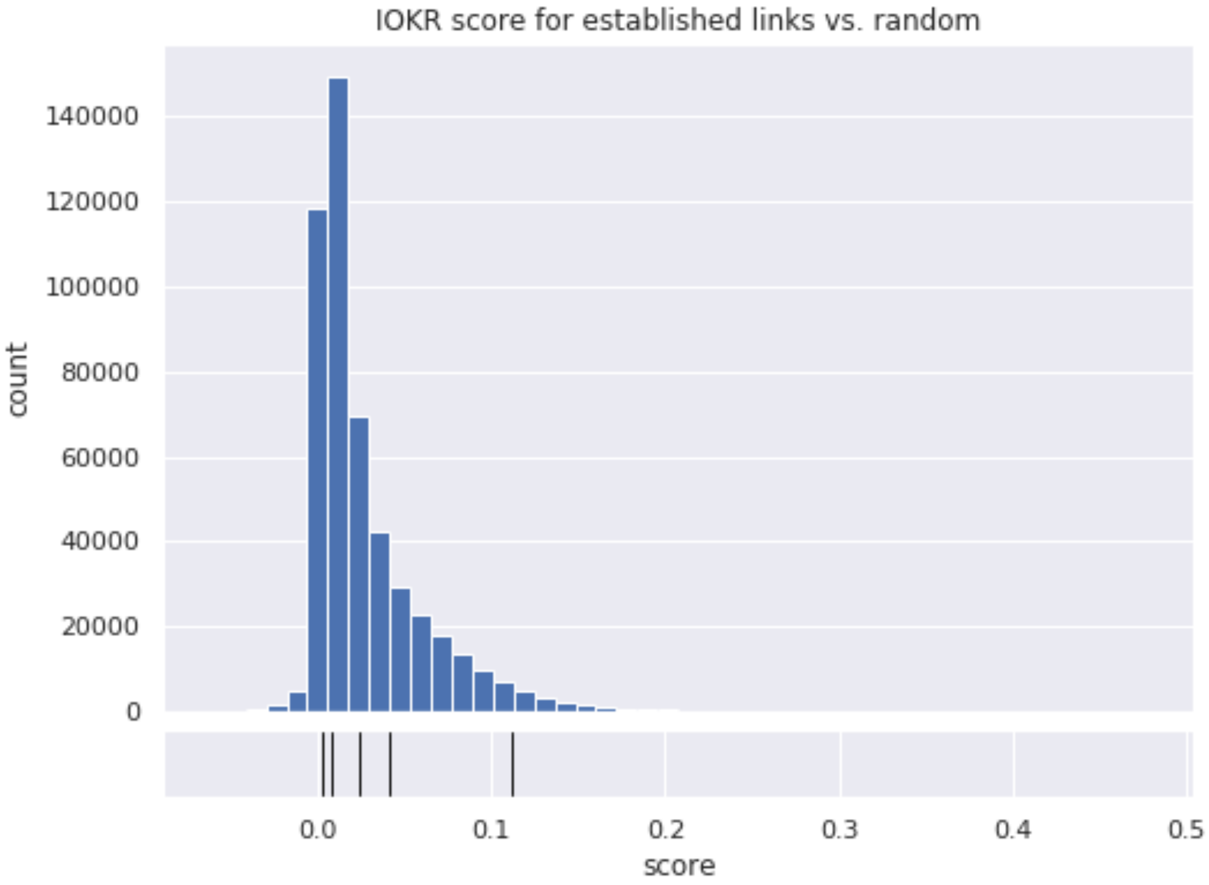

IOKR score for established links vs. random
count
140000
120000
100000
80000
60000
40000
20000
0
0.0
0.1
0.2
0.3
0.4
0.5
score

Gross
