# Supplementary material for: Ranking microbial metabolomic and genomic links in the NPLinker framework using complementary scoring functions
Source: PLoS Comput Biol. 2021 May 4;17(5):e1008920. doi: 10.1371/journal.pcbi.1008920 (PMC8130963; doi:10.1371/journal.pcbi.1008920)
Supplement: S3 Fig — Figures showing the IOKR- and standardised strain correlation scores for the microbial data sets, with the validated links marked. (PDF) [file pcbi.1008920.s004.pdf]

IOKR vs. correlation score

IOKR- (y-axis) and strain correlation (x-axis) scores for all potential links in the three data sets, with histograms of the scores. Verified links are coloured red on the joint plots, and indicated with black lines on the histograms. Verified links are concentrated in the upper-right quadrant, i.e. score relatively high on both axes.

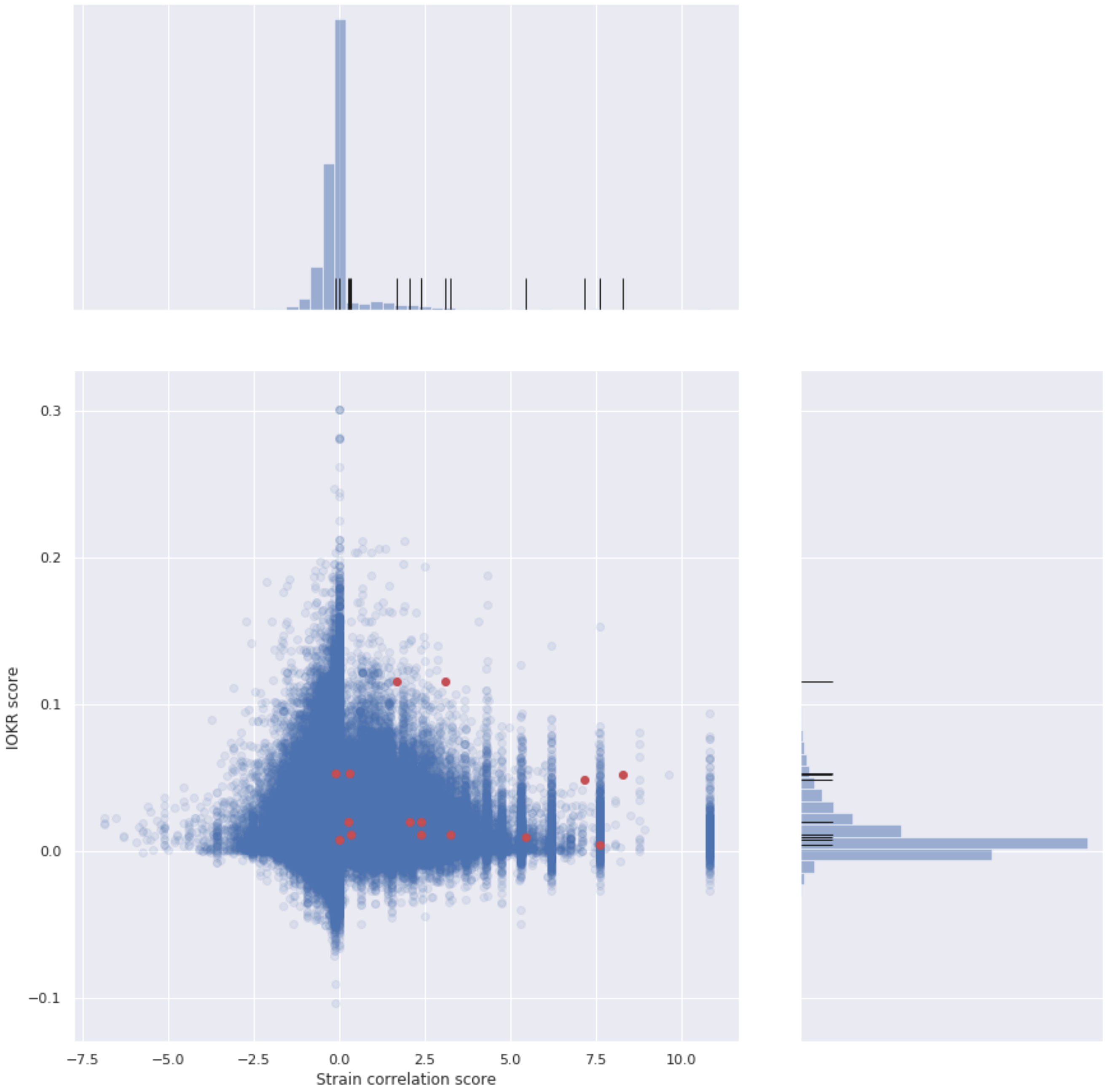

Crüsemann

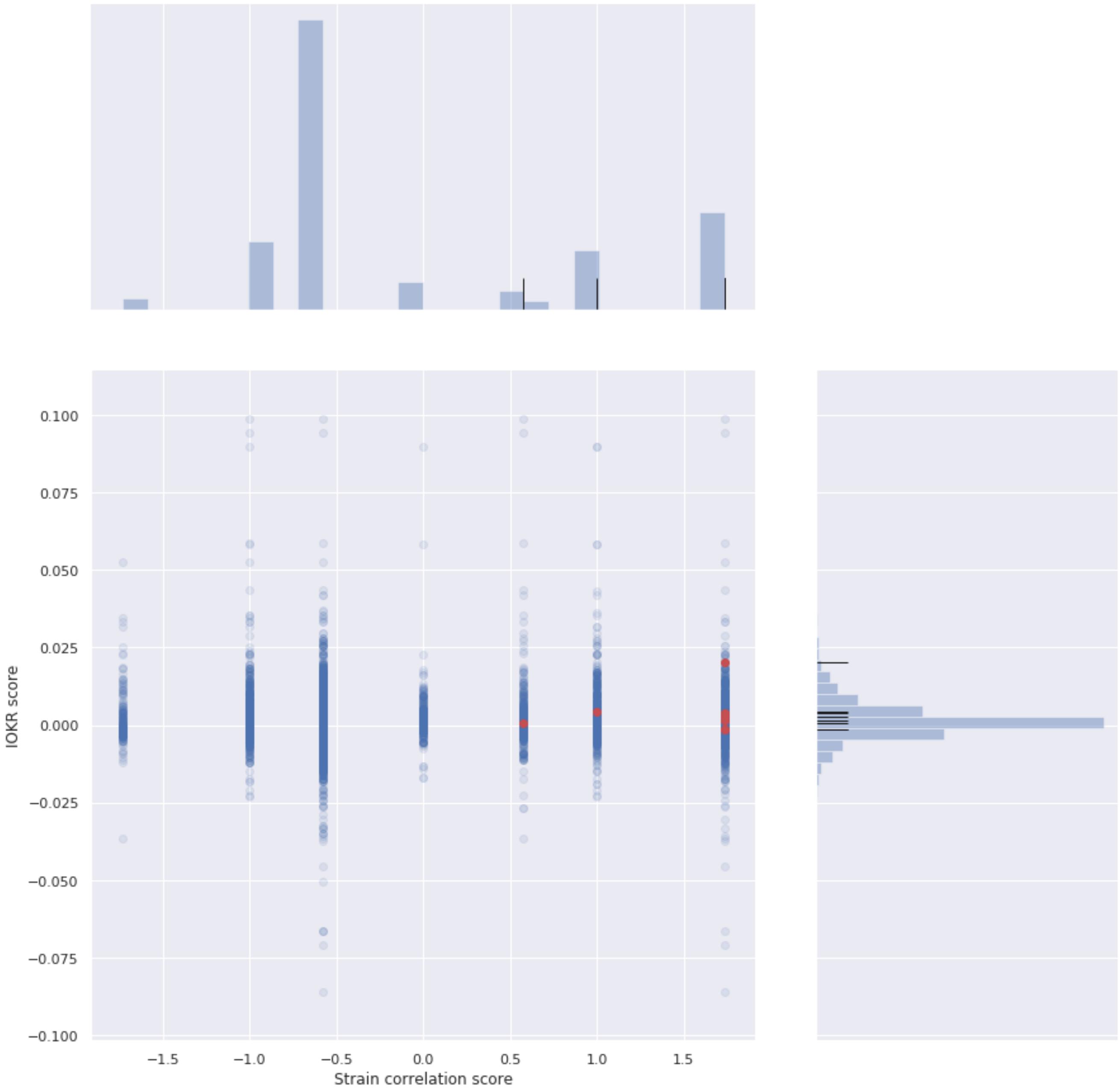

0.100
0.075
0.050
0.025
0.000
−0.025
−0.050
−0.075
−0.100
IOKR score
−1.5
−1.0
−0.5
0.0
0.5
1.0
1.5
Strain correlation score

Leão

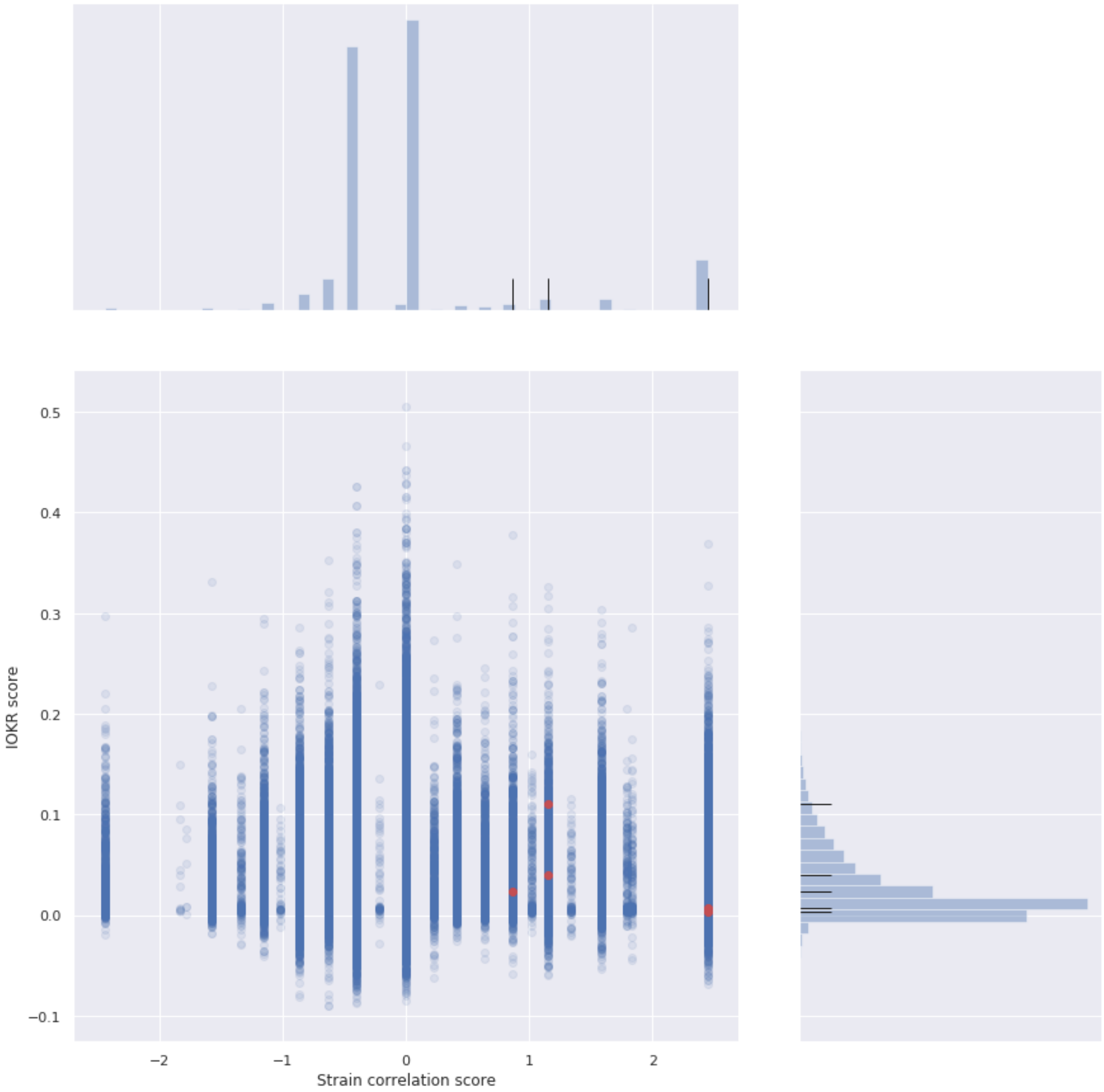

0.5
0.4
0.3
0.2
0.1
0.0
−0.1
IOKR score
−2
−1
0
1
2
Strain correlation score

Gross
