# Supplementary material for: Ranking microbial metabolomic and genomic links in the NPLinker framework using complementary scoring functions
Source: PLoS Comput Biol. 2021 May 4;17(5):e1008920. doi: 10.1371/journal.pcbi.1008920 (PMC8130963; doi:10.1371/journal.pcbi.1008920)
Supplement: S4 Fig — Figures showing the distributions of scores starting from BGCs in validated links for the microbial data sets. (PDF) [file pcbi.1008920.s005.pdf]

Score distributions for a particular BGC

Position of the score for the validated BGC-MF pair (red dot) within the distribution of the scores of the links between that particular BGC and all MFs, for established links (rows). The first three columns show histograms of the raw and standardised versions of the strain correlation score, as well as the IOKR score, for all links including a given BGC, with the score of the correct link indicated. The last column shows the standardised correlation score (x-axis) and IOKR score (y-axis) for the same links, again with the correct link indicated.

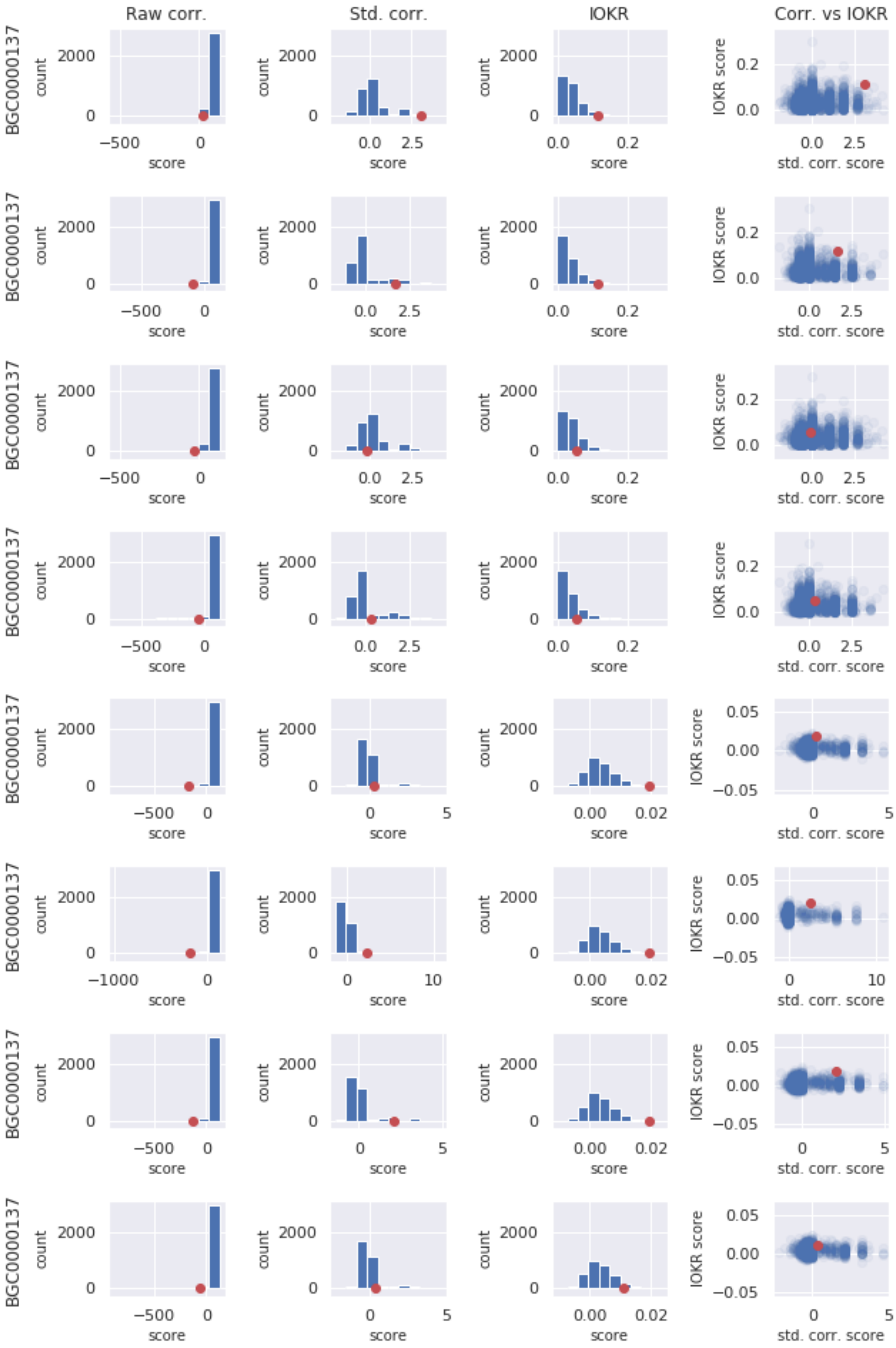

Raw corr.
Std. corr.
IOKR
Corr. vs IOKR
BGC0000137
count
score
IOKR score
std. corr. score

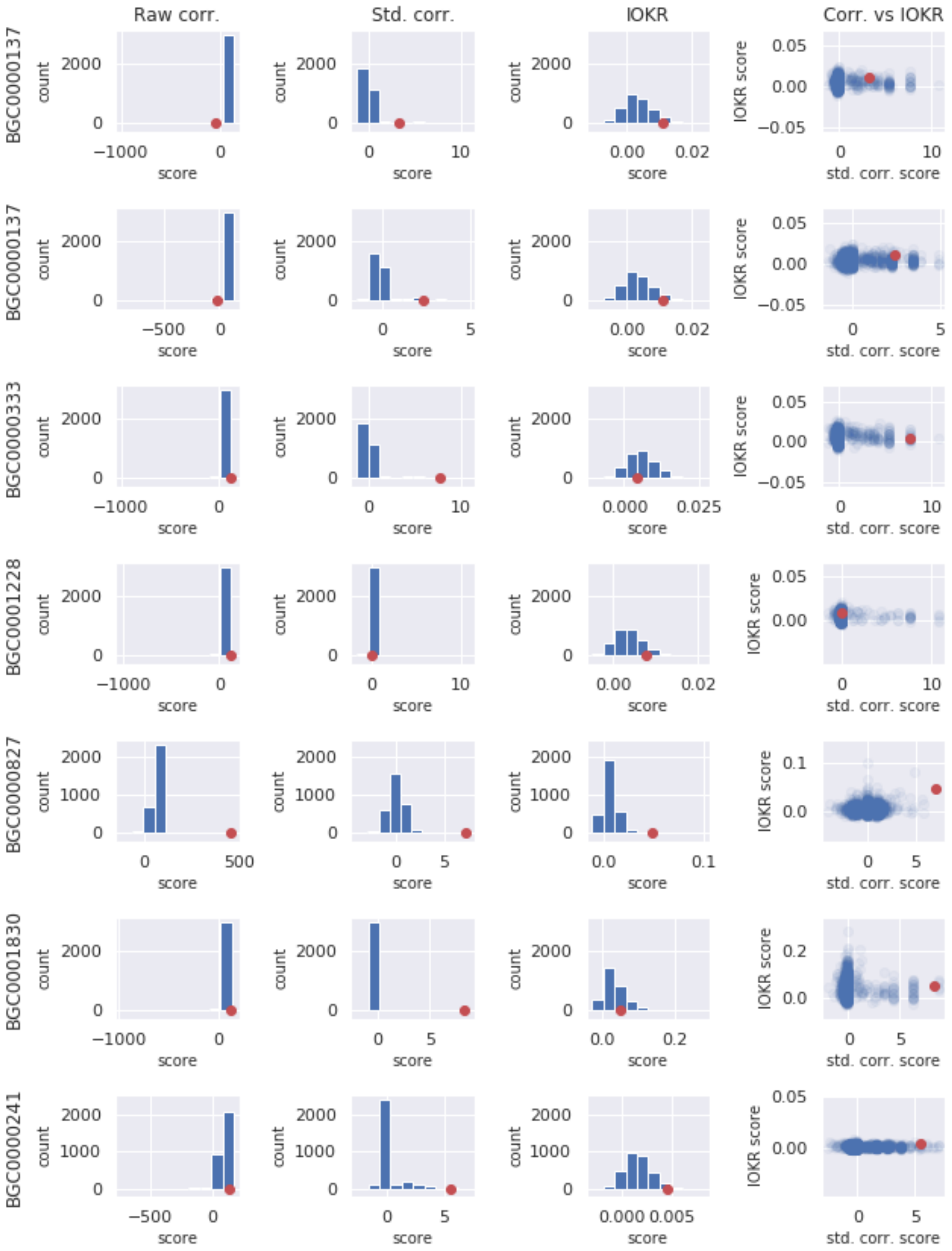

Raw corr.
Std. corr.
IOKR
Corr. vs IOKR
BGC0000137
BGC0000137
BGC0000333
BGC0001228
BGC0000827
BGC0001830
BGC0000241
count
score
IOKR score
std. corr. score

Crüsemann

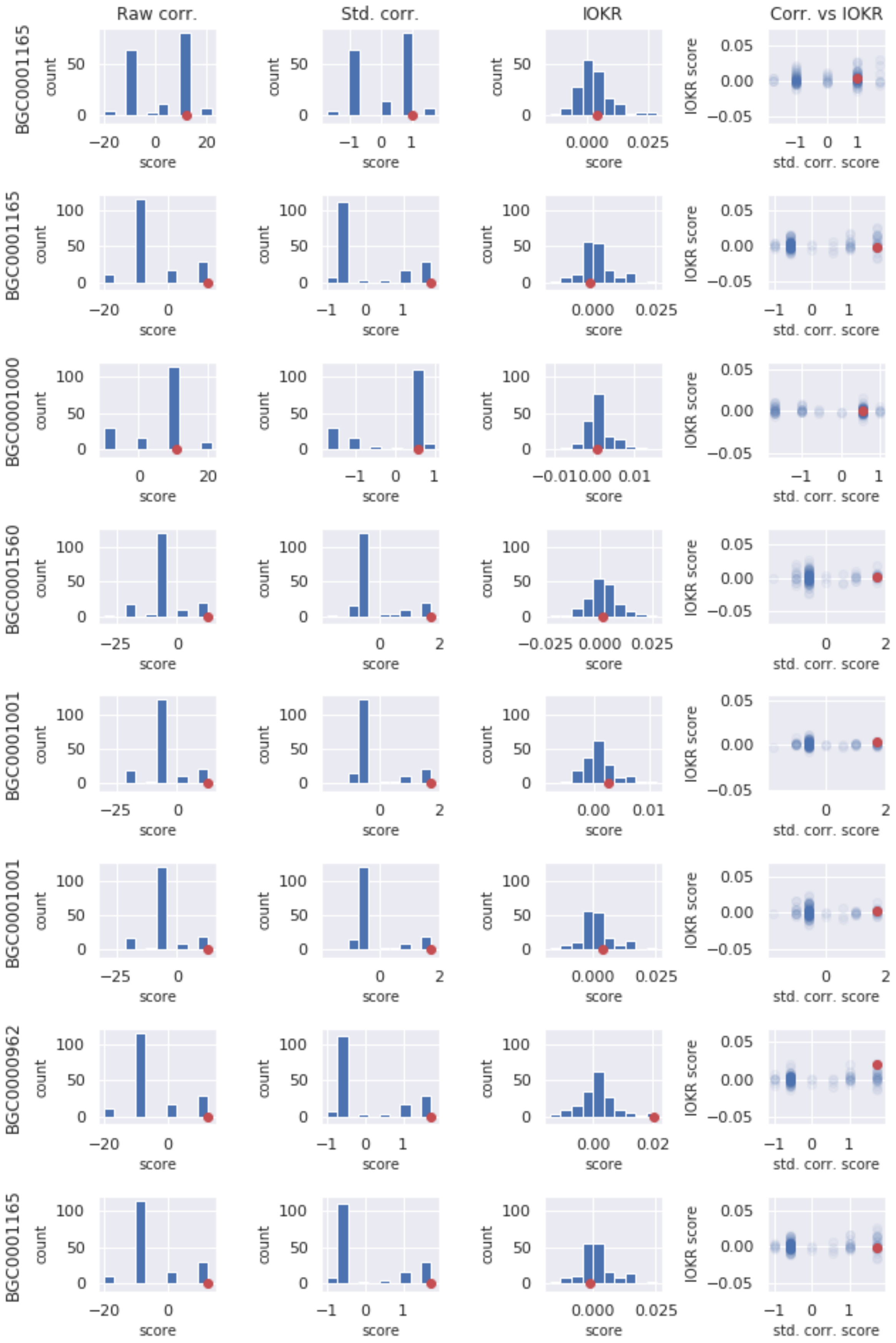
Raw corr.
Std. corr.
IOKR
Corr. vs IOKR
BGC0001165
BGC0001165
BGC0001000
BGC0001560
BGC0001001
BGC0001001
BGC0000962
BGC0001165
count
score
IOKR score
std. corr. score

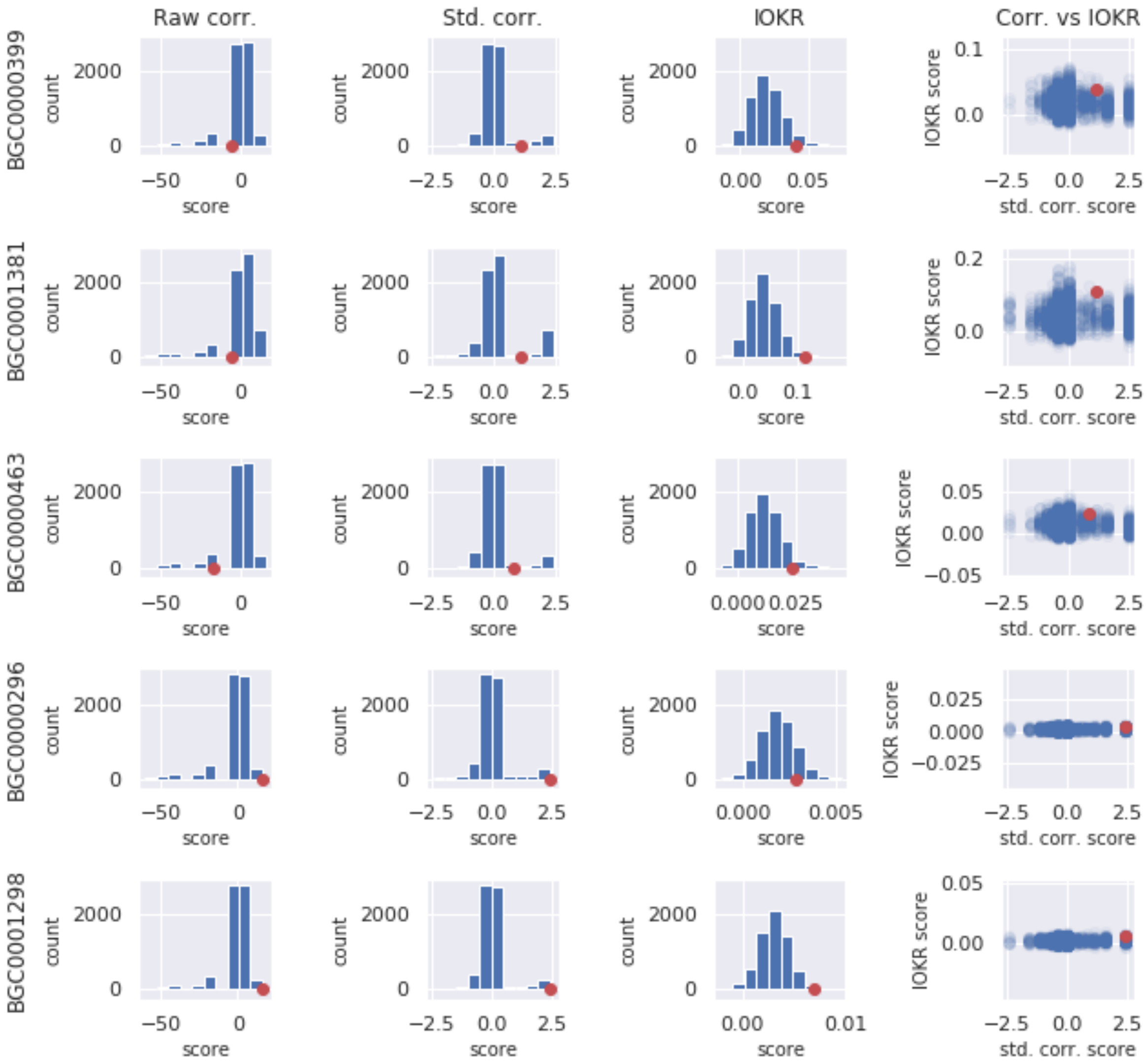
Raw corr.
Std. corr.
IOKR
Corr. vs IOKR
BGC0000399
BGC0001381
BGC0000463
BGC0000296
BGC0001298
count
IOKR score
score
std. corr. score

Gross
