# Supplementary material for: Ranking microbial metabolomic and genomic links in the NPLinker framework using complementary scoring functions
Source: PLoS Comput Biol. 2021 May 4;17(5):e1008920. doi: 10.1371/journal.pcbi.1008920 (PMC8130963; doi:10.1371/journal.pcbi.1008920)
Supplement: Attachment — Submitted filename: nplinker_reply_to_reviewers_20210305.pdf [file pcbi.1008920.s011.pdf]

Dear editor,

We would like to thank the reviewers for their valuable feedback and suggestions. We have taken care to address their concerns in the revised version of our manuscript, and they have undoubtedly helped us clarify our reasoning and improve the manuscript.

While the specific comments from the reviewers are addressed one by one below, we would like to emphasise two general points, which we believe are helpful to keep in mind when considering the comments below and our responses to them.

Firstly, distinction must be made between the NPLinker framework itself, which is a general framework to facilitate mining paired genomic and metabolomic datasets for BGC-spectrum links, and the scoring functions demonstrated here to evaluate the potential links. While we describe one scoring function currently in common use (strain correlation scoring, also sometimes referred to as Metcalf scoring[12]) and introduce two novel ones (standardised strain correlation scoring and IOKR scoring), other scoring functions can quite easily be implemented, as done by Soldatou and co-workers[3].

Secondly, one of the key contributions of the current work is demonstrating in a systematic manner the complementarity of the scoring functions presented. While this complementarity between correlation-based and feature-based approaches has been implicitly used, most obviously when using presence or absence of BGCs in microbial strains as a filtering step to select potential links for further evaluation[4], framing these steps as distinct scoring functions to conclusively demonstrate and quantify their complementarity has not been done before. The performance of the individual scoring functions should therefore also be evaluated in light of this complementarity.

For general ease of reading, we have made the following changes:

- Added numbering to sections for easier reference.
- Added missing heading / section break for Conclusion section

Various typos have been we have fixed throughout the manuscript, and the terms “InChI-string” and “InChI-key” replaced with “InChI” and “InChIKey”, respectively (in keeping with the proper nomenclature).

Line numbers refer to the revised manuscript, while line numbers in parentheses refer to the initial submission.

---

[1] Navarro-Muñoz JC, Selem-Mojica N, Mullowney MW, Kautsar SA, Tryon JH,Parkinson EI, et al. A computational framework to explore large-scale biosynthetic diversity. Nat Chem Biol. 2020;16(1):60–68.

[2] Doroghazi JR, Albright JC, Goering AW, Ju KS, Haines RR, Tchalukov KA,et al. A roadmap for natural product discovery based on large-scale genomics and metabolomics. Nat Chem Biol. 2014;10(11):963–968.

[3] Soldatou S, Eldjárn GH, Ramsay A, van der Hooft JJJ, Hughes AH, Rogers S,et al. Comparative Metabologenomics Analysis of Polar Actinomycetes. Marine Drugs. 2021;19(2). doi:10.3390/md19020103.

[4] E.g. in Goering AW, McClure RA, Doroghazi JR, Albright JC, Haverland NA, Zhang Y,et al. Metabologenomics: Correlation of Microbial Gene Clusters with Metabolites Drives Discovery of a Nonribosomal Peptide with an Unusual Amino Acid Monomer. ACS Cent Sci. 2016;2(2):99–108.

We hope the changes and clarifications presented satisfy both the editors and the reviewers.

Warmest regards,

The authors.

Reviewer 1:

This manuscript by Eldjárn and collaborators describes efforts to create a new bioinformatic tool that more accurately connects biosynthetic gene clusters to specific compounds in a paired metabolomic sample analyzed by tandem MS. This new tool creates a single numeric measure of correlation using two complementary methods: a correlation metric for presence/absence of features, and an analysis of the predicted structural features from the two data sets using a molecular fingerprint strategy (using antiSMASH and MIBiG). This involves a fairly complex algorithm that employs an Input Output Kernel Regression approach. Overall, this seems a valuable new tool for the community, and with attention to the concerns/suggestions below, should be appropriate for publication.

1. Several of the figures are incompletely described in the figure legend such that the precise meaning is not clear. For example, there is no description specifically identifying what the "red dots" are depicting in Figures 6 and 7. The meaning of Figure 8 is also not very clear, and requires a better explanation, including its legend. The authors should go through all of their figures and check that they are fully annotated or described in the Figure legends.

We thank the reviewer for drawing our attention to this omission. Numerous changes have been made to rectify this:

In Figs. 6 and 7, the red dots indicate the validated links. The has been clarified by changing the legend, in Fig. 6 from

"Verified links are marked on the joint plot, and marked on the distribution histograms."

to

"Validated links are indicated in red on the joint plot, and with black lines on the distribution histograms."

and in Fig. 7 from

"Position of the score for the validated GCF-MF pair within the distribution [...]"

to

"Position of the score for the validated GCF-MF pair (red) within the distribution [...]"

Fig. 8: Added to legend "This shows the form of the iso-lines of scores using the \(\ell_p\) function for different values of \(p\) to combine the scores."

2. All of the results in this paper are reported in the aggregate, and therefore, it is quite abstract and theoretical. It would be very useful to show an example or two where an actual BGC is correlated to a specific MS2 spectrum as well as the compound giving rise to these data. That is, it would help to demonstrate the utility of the tool through illustration with a specific example. For example, could the authors find a rare GCF with good BGC predicted

structural insights and then use NPLinker to predict its MS/MS spectra pair. By making sense of the fragments using the predicted structure and the paired spectrum, one can partially/fully confirm the match without the need for isolation and NMR analysis (although a rigorous structure identification would be ideal).

In our current study, most results are presented in the aggregate to allow us to draw robust statistical conclusions. However, some individual results are provided: In particular, Section 3.5 contains results specific to the Crüsemann data set, including ranking information for specific validated links including the actual BGCs and specific MS2 spectra.

As the reviewer rightly suggests, the ultimate goal of the joint analysis of genomics and metabolomics data is to help find links between yet uncharacterized GCFs and MFs. However, a key step in the development of such approaches is demonstrating that these methods will find the validated links present in the Paired Omics Data Platform, as we are doing here.

As we demonstrate in Table 4, for a given initial BGC our ranking methods tend to put the validated link higher in the ranking of potential links for that BGC than the current most common method (the raw strain correlation score), but even so, it is by no means always the highest scoring potential link. Therefore, for novel cases, considerable further effort is needed to actually find and verify the correct link. This would typically include, as the reviewer points out, analytical validation (e.g. NMR), which is beyond the scope of this work (and impossible with publicly deposited datasets for which the original samples are not publically available).

We thank the reviewer for the suggestion of attempting to “explain” the fragments in some links as a means of partly confirming the match. We have attempted this and found some promising results. By fragmenting the metabolites *in silico* (using metfrag) for two of the validated links in the Crüsemann data set, as well as for one high-scoring potential link with a strong input from the IOKR score, we could assign peaks in the MS2 spectrum to peaks in the hypothetical spectrum, representing specific substructures of the metabolite. By excluding some of these peaks from the MS2 spectrum, we could observe how the exclusion changed the IOKR score for the link, indicating how relevant the IOKR scoring function considers the inclusion or exclusion of this particular substructure and thus providing insights into its validity and working.

In particular, for rosamicin, which is a validated link, we could identify the peak representing an aminosugar subunit of the metabolite as having a strong influence on the ranking of the metabolite, with the exclusion lowering the ranking from 16th highest to 163rd highest.

Details of the experiment have been added to the Supplementary Information, and a paragraph has been added to Section 3.3, l.500-505, to reflect this:

Furthermore, the NPLinker framework has already been used by Soldatou and co-workers to putatively link the metabolites ectoine and chloramphenicol to their producing BGCs[5]. The first paragraph of Section 2.6, l.263-267, has been changed to reflect this:

“To facilitate analysis of paired genomics and metabolomics data sets, we developed NPLinker, a Python module to accelerate and support the process of automatically linking GCFs or their BGCs with observed mass spectra. Demonstrating its utility, NPLinker has been used by Soldatou and co-workers to putatively link the known metabolites ectoine and chloramphenicol to their producing BGCs [31].”

“As an additional level of validation, we tested some high scoring links by exploring whether it was possible to manually match peaks in the MS2 spectra to the chemical structures. We then assessed how much these peaks influenced the ranking produced by IOKR. If peaks that can be matched to chemical structures have a large influence on ranking, it provides additional evidence to the validity of the links. We found several examples of this in both validated and prospective links, details of which are provided in the Supplementary Information.”

3. Why is “ranking links” used in the title instead of “connecting” or “linking” BGC to MS/MS? Additionally, it is suggested to refine the wording to “connecting known BGCs to cryptic MS/MS spectra” to make it clear to the reader what the tool can do and not do, and how it can be applied. It’s important to point out to the reader that this tool is not useful for classifying non-homologous/unknown BGCs.

We see “ranking” as a key step towards “connecting” or ”linking”. I.e. for, say, a particular BGC, a useful output from a tool such as this is a list of potential spectra, ranked by one or more scoring functions. This ranked list can then be subjected to further validation. To be able to pluck a single candidate out of the data (as we believe would be suggested by “connecting”) is currently not possible (and will likely remain so for the foreseeable future). Actually, NPLinker is meant to facilitate this process though and through adoption of novel scores as presented here and elsewhere[6], we expect that the number of highly-ranked links to sieve through will decrease in the coming years.

Regarding the limitations of the tool, NPLinker itself is not limited to known BGC or spectra. On the other hand, the various scoring functions that it implements might be limited to some subset. For instance, while the strain correlation score requires no information apart from strain membership patterns for the GCFs and MFs, the IOKR scoring function is at present limited to BGCs for which molecular structure has been predicted (but it does work with all spectra). Using predictions of molecular properties, such as amino acid content, one could easily imagine a scoring function that only works for a particular product type, such as NRPS.

---

[5] Soldatou S, Eldjárn GH, Ramsay A, van der Hooft JJJ, Hughes AH, Rogers S,et al. Comparative Metabologenomics Analysis of Polar Actinomycetes. Marine Drugs. 2021;19(2). doi:10.3390/md19020103.

[6] Soldatou S, Eldjárn GH, Ramsay A, van der Hooft JJJ, Hughes AH, Rogers S,et al. Comparative Metabologenomics Analysis of Polar Actinomycetes. Marine Drugs. 2021;19(2). doi:10.3390/md19020103.

l.287 (l.258): Added a paragraph to Section 2.6 (NPLinker) to clarify the distinction between the framework and the scoring functions, and to emphasise that the restriction to known BGCs is a function of the scoring function, not NPLinker. The next-to-last paragraph now reads:

"NPLinker creates a set of hypothetical links between the metabolomic and genomic objects, which can then be evaluated using various scoring functions, both scoring functions that are supplied with NPLinker and custom scoring functions. Not all scoring functions need to be defined for all potential links. For instance, in its current formulation, the IOKR scoring function described here can only rank links involving BGCs that have predicted molecular structures, which in practice means BGCs with significant homology to known BGCs, whereas the strain correlation score can rank any GCF-MF link. Other scoring functions may for instance only apply to particular product types. Fig. 4 shows how NPLinker combines the various data sources."

l.46 (l.47): Emphasise that we're using ranking of all links as a step along the way to linking BGC to MS/MS. Changed

"Computational approaches have therefore concentrated on scoring possible links, and using the resulting ranking to prioritise links for further analysis."

to

"To link BGCs to MS2 spectra, computational approaches have therefore concentrated on scoring this collection of all possible links, and then ranking the links by score. Such approaches can prioritise links for further analysis, in order to accelerate the process of finding the correct links in the set of all possible links."

4. Part of the validation set from Paired Omics DB includes metagenome-assembled genomes (MAGs). MAGs tend to have two issues: fragmentation, splitting BGCs into multiple contigs, and; contamination, maybe adding extra BGCs to the MAG. Regarding fragmentation, suggest checking if the test BGCs are on the edge of contigs or if they are considered complete. For contamination, suggest checking for each BGC in a strain if the GC content deviates too much from the average. For more detailed inspection, tetranucleotide signature and closest relative taxa from the database are also options to confirm that the BGCs are not from contaminants. A Table for the 22 BGCs in the test set from Paired Omics Data Platform (PoDP) would be ideal, containing at least BGC type, completeness and GC deviation. This table could be part of the supporting information.

While some of the predicted BGCs could be either fragmented or due to contamination, this is not the case for the validated BGCs, since they are sourced from papers where they have been rigorously verified by either existing literature, the submitters of the datasets, or both.

The bias due to fragmented or contaminated BGCs would therefore manifest as a higher number of non-valid potential BGC-MF links, and not affect the evaluation of the scoring function, and in particular not the comparison with the strain correlation function currently used: assuming that the validated links are accurate, this noise level should affect all the scores, including the (baseline) strain correlation score, and so shouldn't affect the

performance improvement. Thus we do not think that analysing the quality of the MAG-BGCs would add much to the main conclusions of our paper.

5. Why do the authors use BiG-SCAPE if BiG-SLICE scales are much better? It would be interesting to check how the algorithm behaves with BiG-SLICE vs BiG-SCAPE.

While BiG-SLICE performs better on large data sets, the trade-off is a loss in precision - in fact, the authors of BiG-SLICE recommend BiG-SCAPE for more detailed analysis. As the data sets considered in this paper are small enough that computing time isn't a limiting factor, we prefer the higher accuracy afforded by BiG-SCAPE.

l.67 (l.66): Added reference to BiG-SLICE, explaining the preference for BiG-SCAPE:

"The current state-of-the-art tools for microbial BGC clustering are BiG-SCAPE [13] and BiG-SLICE [21]. Of the two, BiG-SCAPE offers higher precision, and as the data sets under consideration here are not of the size that performance becomes a problem, BiG-SCAPE is used for BGC clustering throughout this article."

6. Why have the authors selected only three datasets out of 70 public datasets with over 100 validated links? The testing size of 22 BGCs is very small and may not represent many other microbes.

While the Paired omics Data Platform does contain a much larger number of data sets, only a few of these contain detailed annotations for the BGC/MS2 links. Of the ones that do, the Crüsemann data set has by far the largest number of strains, making the strain correlation score particularly informative for that data set. In fact, at the time the experiments described in the current work were done, the Crüsemann data set was the only one with both a sizable strain population and a number of thoroughly annotated links. Correlation scores for datasets including only a handful of strains are unlikely to yield much information.

l.389 (l.320): Added an explanation for why only a limited subset of the data sets in PoDP are suitable for our purposes:

"Although the platform contains a large number of data set with validated links, the minority of these contains detailed links between particular BGCs and MS2 spectra, and fewer still of these contain a large enough number of strains for the strain correlation score in particular to carry sufficient information."

7. Perhaps it would be good to show that the tool outperforms some other straight forward ways to match predicted structures with MS/MS spectra; for example, matching via Tanimoto scores of predicted structures, one can collect predicted structures for the queried spectra using in silico tools and match those to predicted structures from tested BGCs. Alternatively, matching via direct presence/absence using Jaccard index, one could create a matrix of presence/absence for GCFs and a matrix for MFs and then make links between rows using Jaccard index. NPLinker should be able to generate more correct links than these other methods. Benchmarking NPLinker to other methods is an important recommended revision to this manuscript.

While we agree that benchmarking the scoring functions presented here against other scoring functions is important, the current study already contains extensive comparisons against the current state-of-the-art, which is the strain correlation score defined by Doroghazi et al[7]. These can be found in Section 3.1, Table 2, where we demonstrate how standardising the strain correlation score improves its performance, i.e. given a subset of validated links in the set of all potential links, the standardised strain correlation score ranks the validated links higher, relative to all potential links, than the strain correlation score does without standardisation.

Furthermore, it is worth pointing out that the NPLinker framework is in no way limited to the scoring functions described in the paper, and as long as they can be converted into numerical scores, any other methods can be incorporated into NPLinker alongside or replacing the scoring functions described here.

The scoring functions suggested could be implemented in the NPLinker framework, either complementing or replacing the current scoring functions. However, we consider this to be outside the scope of the current study.

The latter half of the NPLinker section (Section 2.6) has been rewritten to emphasise that the NPLinker framework is independent of the scoring functions used to evaluate the hypothetical links:

"NPLinker creates a set of hypothetical links between the metabolomic and genomic objects, which can then be evaluated using various scoring functions, both scoring functions that are supplied with NPLinker and custom scoring functions. Not all scoring functions need to be defined for all potential links. For instance, in its current formulation, the IOKR scoring function described here can only rank links involving BGCs that have predicted molecular structures, which in practice means BGCs with significant homology to known BGCs, whereas the strain correlation score can rank any GCF-MF link. Other scoring functions may for instance only apply to particular product types. Fig. 4 shows how NPLinker combines the various data sources."

8. It is concerning that the accuracy seems very low (12 to 29% in Table 3?) and the authors should make a comment on it. An indication of accuracy should also be discussed or provided in the abstract and in the introduction. Table 3 should also have top-n accuracy for raw correlation, standardized correlation, standardized correlation plus IOKR, instead of only for IOKR.

While accuracy of 12 to 29% appears low, the problem of matching spectra to metabolites is a very difficult one, and achieving top-1 accuracy above 20% is highly non-trivial, unless the candidate set in the space of metabolites is heavily restricted a priori.

---

[7] Doroghazi JR, Albright JC, Goering AW, Ju KS, Haines RR, Tchalukov KA,et al. A roadmap for natural product discovery based on large-scale genomics and metabolomics. Nat Chem Biol. 2014;10(11):963–968.

Computing the Top-n accuracy for the strain correlation scores using the MIBiG/GNPS data set, as in Table 3, is impossible. This is because strain correlation scoring (standardised or not) relies on an underlying population of strains from which both the genomic and metabolomic data is drawn. This is not available for the GNPS library spectra.

l.480 (l.382): Added text to explain that the accuracy for this kind of problem is generally quite low:

“Matching spectra to a set of candidate molecules is a very hard problem, and achieving top-1 accuracy above 20% is highly non-trivial, unless the candidate set is heavily restricted. For instance, the single best-performing kernel used by Brouard and co-workers in [27] achieved around 20% top-1 accuracy using cross-validation on the GNPS training set, but with a different choice of candidate set for each spectrum, and a better curated set of spectra.”

9. GNPS has 38,102 spectra with annotated SMILES; why not use it all instead of only 4,138?

There are two, related, reasons for this. This subset represents high-quality, thoroughly verified MS2 data, collected with similar device configuration. Secondly, this subset has been used in numerous previous articles describing machine learning methods for MS2 analysis, making comparisons between different models easier.

l.305 (l.268): Added explanation of the choice of a subset of GNPS spectra:

“While GNPS contains a far higher number of spectra with structural annotations, this subset represents high-quality MS2 data with similar device configuration and thoroughly verified structural annotations. Furthermore, this same subset has been used in numerous previous articles (such as [27] and [28]), making performance comparisons between different models easier.”

10. “Assuming that similar BGCs give rise to similar compounds, we used this list in turn to assign one or more molecular structures to BGCs, according to how many high-scoring matches are found in MIBiG (or none, if no match is found).” It’s a risk assumption because a single gene difference can cause a profound structural change or no change at all. The “linear” InChI keys are a good way to match MIBiG BGC to GNPS MS/MS. However, aligning the BGC labels with the MS/MS labels is suggested in order to double check that the links are correct.

Thanks for the suggestion, as a sanity check we compared the GNPS- and MIBiG-reported names of the metabolites. While they were occasionally different, in all of the cases where the name reported in GNPS could easily be resolved to a metabolite name, the difference in naming could be explained.

While we appreciate that a change of a single in a BGC can cause major structural changes, the assumption that similar BGCs produce similar molecules is fundamental to the whole concept of clustering BGCs into GCFs. In fact, one could argue that a good similarity function on the space of BGCs should be able to take into account how likely a difference of

a particular gene (in particular situation) is to make a considerable difference in the molecular structure.

In this paper, we do not directly work with the similarity functions between BGCs, but use the clustering of BGCs into GCFs provided by BiG-SCAPE. This does, however, implicitly rest on the similarity function defined in BiG-SCAPE.

l.339 (l.282): Added a section on why we need this assumption (similar BGCs yield similar metabolites):

“While small changes in domain composition can potentially result in a big change in molecular structure, the fundamental premise of clustering BGCs into GCFs rests on the assumption that the distance between the BGCs is defined in such a way that the assumption above is true, at least for the majority of cases. However, verifying the validity of this assumption, and therefore the validity of the distance functions, is outside the scope of this paper.”

11. Cite Metaminer instead of RippQUEST.

We thank the reviewer for pointing this out.

l.59 (l.58): Changed citation to MetaMiner

12. Why is the standardized score for one of the family pairs in Fig. 2 A zero? If that’s the case, it requires more explanation.

The standardised strain correlation score for the top pair in Fig. 2 A is zero because the GCF contains all the strains in the population. Therefore, its overlap with the strain set of any MF is complete. In fact, this is a key strength of the standardised score. Without standardisation, this would give a very high score. In reality a BGC present in all strains is unlikely to be of interest.

l.166 (l.159): Added an explanation of a standardised strain correlation score of zero in terms of expected overlap:

“A standardised strain correlation score of zero means that the degree of overlap between the two strain sets is the same as would be expected if they were chosen at random, given the size of the GCF and the MF. For instance, since the GCF in the example contains all the strains in the population, any MF will have complete overlap with the GCF, giving all links for this GCF a score of zero, which is actually what you would like to see for such links given their very low chance to be informative”

13. On page 11, lines 332-339, please indicate if there was a similarity threshold for a “known cluster blast” match to be consider a true annotation?

We thank the reviewer for pointing out this omission. The similarity threshold for the “known cluster blast” matching was a cumulative BLAST score of 10000. This filtering was required

to reduce the noise from low-quality MIBiG hits, but the annotations appeared to be stable with regard to the precise value of the cutoff.

l.409 (l.336): Added info about ‘known cluster blast’ score threshold:

“To avoid excessive noise due to low-quality matches, the cutoff for this matching was a cumulative BLAST score of 10000, but the data sets being analysed did not appear very sensitive to variation in this value.”

14. What is the difference between validated vs verified links? If these are being used as synonyms, the authors should select one and only use one to avoid confusion. For instance, the use of these two terms as column headers in Table 4 is very confusing.

We appreciate the suggestion and have changed the terminology to ‘validated links’ in all cases.

15. In Table 4, does the last column indicate that in none of the cases the correct response was the first link? Or is that the ranking of the validated link? Does the term “higher” refer to a ‘better score’ or a ‘bigger number’ (which would have opposite meanings and conclusions)?

We agree that the use of the term ‘higher ranking’ can be ambiguous - a “high ranking” can be taken to mean a high index in the ranked list (bad) or a position near the top of the list (good). We have gone through the manuscript to try to clarify this.

The term ‘higher’ in Table 4 refers to ‘higher score’, as stated in the heading: the full column heading is “no. of links with higher \sigma_IOKR and \sigma_corr than the validated link”.

The last column of Table 4 is the position of the validated link in the ranking, same as the rest of the columns, i.e. if no other links are ranked higher, the link is at 1. The table notes have been changed to reflect this. This ranking is known as the Product Order, as described below.

Table 4: Changed row label to refer to “best” rank, rather than “lowest” rank.

l.591 (l.487): Added a paragraph describing the Product Order, and explaining why it’s not particularly useful in this case, despite yielding the highest ranking of validated links:

“The last column of Table 4 shows the rank of the validated links in the product order. While resulting in the highest rank of the validated links, in practice the product order is not particularly useful as it yields a very high number of links that are not comparable using the relation, i.e. all links a, b where σ_IOKR(a)< σ_IOKR(b) but σ_corr(b)< σ_corr(a), or vice versa.”

16. Page 2, line 12 – spelling error ‘unknown’

Thanks for spotting this, it has been fixed along with various other typos.

17. P3, l90 – change “on” to “an”

l.94 (l.90): Removed ‘on’. Sentence now reads:

“[...] we propose the use of Input-Output Kernel Regression for this task.”

18. P10, l288-290 – it’s not clear why these number do not total up? Is it that there are redundant spectra, and many redundant BGCs, such that there are many fewer “unique” spectra or BGCs compared to the number of pairs? This needs some further explanation.

The relationship between MIBiG BGCs and GNPS spectra is many-to-many: Each MIBiG BGC can have one or more metabolites, which can have one or more spectra in GNPS. Conversely, the same metabolite can have multiple entries in MIBiG, for instance when the same metabolite is produced by different organisms.

l.355 (l.291): Clarified that the MIBiG/GNPS relation between BGCs and spectra is many-to-many:

“As MIBiG can contain more than one BGC encoding a given metabolite, the relation between spectra and BGCs in this list is many-to-many: some MIBiG BGCs can encode multiple metabolites, each of which can have multiple spectra in GNPS. Conversely, more than one MIBiG BGCs may encode the same metabolite, or different BGCs may encode metabolites that have identical first parts of the InChIKey, and are therefore linked with the same spectra.”

19. P18, l533 – the ending sentence seems unnecessarily limiting. Three types of specific biological activity are noted, but there are of course many other pharmaceutical classes wherein new medications are needed. A more general statement seems more appropriate, such as “…. novel chemistry that is much needed in diverse areas of human medicine and agriculture”.

l.646 (l.533): Changed

“[...] novel chemistry that is much needed to fight off pathogenic bacteria, viruses, and insects.”

to

“[...] novel chemistry that is much needed in diverse areas of human medicine and agriculture”.

Reviewer #2: The manuscript titled “Ranking microbial metabolomic and genomic links in the NPLinker framework using complementary scoring functions” by Eldjárn et al demonstrates a ranking scheme to score link the genomic and the metabolomic data. Specifically, the authors use the information about strains, biosynthetic gene clusters (BGCs) and the metabolic fingerprints (MFs) obtained from mass spectra. The authors raise concerns over the existing scoring scheme and propose a method to modify this score to circumvent the challenges. Besides, the authors propose the use of an Input-Output Kernel Regression (IOKR) to predict the ranking of BGCs by combining the information from strains and MFs. Further, they integrate the scores obtained from the above two proposed methods and suggest that the combined score is better. Overall, I find the method reasonable; however, the proposed method requires further validations. Currently, the validation relies heavily on the predictions from antiSMASH, although there are other BCGs tools available. Further, at some places in the manuscript, I observe that the scores obtained using IOKR are lower, raising critical questions about the method proposed. I have listed my suggestions below.

Major

1. Lines 158-159 - What do the correlation scores indicate? What is the range of the correlation scores?

The standardisation of the strain correlation score involves shifting the score and scaling it so that it has a mean of 0 and standard deviation 1. The actual range is not specified, but is dependent on the population size, in that the resolution of the tails (upper and lower) puts a limit on how high the scores can be. Conversely, the range of the raw strain correlation score is very much dependent on the population size, and a priori, evaluating the mean of the distribution for that score is very difficult.

l.162 (l.158): Specified the mean and the standard deviation of the strain correlation score:

“While still not having an upper or lower bound, the standardised correlation score has mean 0, and variance 1.”

2. Lines 176-177 - For compounds having similar structures, how is the mapping done from MS2 spectra to the space of metabolites? In such a case of very similar compounds, how would the scores vary? How do the authors handle the mass spectra of cyclic compounds and stereoisomers?

For the IOKR scoring function, the mapping from MS2 spectra to the space of metabolites (the horizontal arrow in Figure 3) is never explicitly constructed. The mapping from MS2 spectra to the space of Molecular Fingerprints is learned from the MS2-metabolite pairs in the GNPS library training data. Therefore, IOKR can only learn those features of the molecule that a) can be observed in the spectra, and b) are present in the training set.

This problem of generalising to features not seen in the training set is a drawback common to all ML approaches. In that sense, our approach handles all spectra (including those of cyclic peptides and stereoisomers) in the same way. It has to be noted that MS/MS spectra can rarely differentiate between stereoisomers, which is a general and well-known

disadvantage of mass spectrometry-based characterization. In fact, linking to the genome could help to assign stereocenters if those are clearly present in the genetic machinery.

l.186 (l.175): Added text to emphasise that the mapping from spectra to fingerprints is learned from the correspondence between the MS2 spectra and molecules in the training set, rather than explicitly constructed. This means that IOKR is only able to distinguish between molecules based on features it can observe in the spectra - i.e. in the case of stereoisomers, for instance, IOKR is highly unlikely to be able to make out any difference at all. The relevant paragraph now reads:

“In principle, IOKR works by first learning a mapping from the space of spectra to the space of molecular fingerprints. This mapping, along with the function mapping molecular structures to their molecular fingerprints, is then used to project similarities in the input space (spectra) and the output space (molecular structures) to the molecular fingerprint space. This allows searching a candidate set of metabolites for the element giving the closest match in the space of molecular fingerprints to the predicted fingerprint for the spectrum.”

3. Lines 212-213 – Since the input spectra are filtered to include only the peaks in training data, wouldn’t there be a bias?

It is common practice for *in-silico* metabolite identification approaches to apply some filtering to the input spectra. We have used the training data used by Brouard et al[8] in which peaks were filtered depending on whether or not they appear in the predicted fragmentation tree for the spectrum. Post-training, peaks that were not seen during the training phase are not used by the predictive model to make predictions and thus cannot provide any information. This is a characteristic of all ML approaches: features not observed in training cannot help in prediction. It is a downside of ML approaches. Despite this, ML approaches still provide state-of-the-art performance in this task.

l.229 (l.212): Added justification of the filtering approach:

“While this in theory might bias the model towards the training set, in practice this approach is widely used. The training set is large enough that it contains enough ions to build a robust model, and the effect of the bias are small, as borne out by comparing this filtering approach to the filtering approach used by PPKr in [27], which filters peaks based on proposed fragmentation trees.”

4. It is quite unclear from the methods how the authors link the mass spectrum with molecular fingerprints. I suggest that the authors explain this further in the manuscript.

For the training set, the spectra from GNPS have assigned molecular structures which can be used to compute the molecular fingerprints.

[8] Brouard C, Shen H, Dührkop K, d’Alché Buc F, Böcker S, Rousu J. Fast metabolite identification with Input Output Kernel Regression. Bioinformatics.2016;32(12):i28–i36.

For prediction, the spectra to fingerprint mapping is performed by the IOKR model, which is trained on annotated training data. We have added some brief sentences to this effect and pointed readers more clearly to the original IOKR paper where it is described in more detail.

l.186 (l.175): Emphasise that the mapping between MS2 and fingerprints is learned from the training data. Paragraph now reads:

“In principle, IOKR works by first learning a mapping from the space of spectra to the space of molecular fingerprints. This mapping, along with the function of sending molecular structures to their molecular fingerprints, is then used to project similarities in the input space (spectra) and the output space (molecular structures) to the molecular fingerprint space. This allows searching a candidate set of metabolites for the element giving the closest match in the space of molecular fingerprints to the predicted fingerprint for the spectrum.”

l.207 (l.194): Add a sentence emphasising that the mapping between MS2 and fingerprints is learned from annotated training data:

“These can be predicted from the MS2 spectra, using a function learned from annotated training data, and calculated from structural predictions for the BGCs. Notably, IOKR is not limited to particular product classes, as it requires only spectra and predicted molecular structures.“

5. It is also not clear how the problems reported in lines 223-235 are alleviated by combining the two techniques since Table 1 does not report that scores obtained from IOKR+standardized correlation.

While Table 1 does not report the scores obtained from IOKR+standardised correlation, the relationship between the two is discussed in detail in Sections 3.4 and 3.5. Table 1 is simply intended to demonstrate that the validated links are generally assigned higher scores than others using the standardised strain correlation- and IOKR scores.

Combining the IOKR and standardised correlation scores is not a straightforward thing to do. For example, there is no guarantee that the scores are comparable, because they are not necessarily on the same scale. In Sections 3.4 and 3.5, the performance of the scores is evaluated in much more detailed ways than by comparing the means of the distributions. Section 3.5 also includes discussion on how the scores can be combined, including the sum of the two scores, which corresponds to the l_1 norm.

Added note to Table 1 explaining that the scores are not guaranteed to be directly comparable:

“Note that the scores are not guaranteed to be on the same scale, so they are not directly comparable.”

l.257 (l.233): Added reference to Sections 3.4 and 3.5, where the complementarity of the scores is demonstrated and they combined for ranking purposes. The paragraph now reads:

“We demonstrate in Section 3.4 that combining the two approaches by looking at potential links that have a robust ranking using both scoring functions increases the ratio of previously validated links to all links in the joint top percentiles compared to using either one of the approaches. Furthermore, Section 3.5 introduces concrete ways of combining the scores to make use of this complementarity.”

6. Table 1 – Scores of the links reported by IOKR offers very little improvement over the standardized score – in which case, what is the novelty behind using IOKR? I believe it is important for the authors to discuss this in the main manuscript.

The novelty behind using IOKR is two-fold: It complements the strain correlation score, and it is not product-type dependent.

One of the main advantages of IOKR is its complementarity with the strain correlation score. Therefore, it is not being proposed to replace it. As discussed in Section 2.5, the strain correlation score and IOKR score are based on very different properties of the links, and can therefore each prioritise links that the other misses. This complementarity is demonstrated in Sections 3.4 and 3.5.

In addition, unlike most feature-based scoring functions, IOKR is not product-type dependent.

Both are present as bullet points in the introductory section (points 2 and 3). Complementarity is discussed in section 3.4: Complementarity of IOKR and strain correlation scoring.

Added note to Table 1 explaining that the scores are not guaranteed to be directly comparable:

“Note that the scores are not guaranteed to be on the same scale, so they are not directly comparable.”

l.209 (l.194): Added text to reinforce the message that IOKR is independent of product type:

“Notably, IOKR is not limited to particular product classes, as it requires only spectra and predicted molecular structures.”

7. Lines 372-376 is quite unclear – What is a correct metabolite? Suggest rephrasing for clarity.

We appreciate the suggestion to clarify this paragraph, it has been rewritten. Lines 465-474 now read:

“The MIBiG/GNPS data set consists of sets of associated BGC, metabolite and spectrum. As implemented in [27], IOKR gives a ranking of links between spectra and metabolites, in our case using as a candidate set all metabolites that occur in the data set. To find the ranking of the correct metabolite match for a given spectrum, we take the rank of the highest-ranking metabolite that is associated with the spectrum. To turn that ranking into the ranking of the

correct BGC for the spectrum, we take the set of metabolites that are ranked higher than that metabolite, and the set of BGCs associated with any of those metabolites. The rank of the correct BGC is the number of BGCs that are associated with a metabolite that is ranked higher than the correct metabolite highest-ranking metabolite associated with the spectrum."

8. Line 374 – How are the rank ties resolved?

Because we are only looking for the best ranking of a particular link, the ranking process does not allow for ties of correct matches. This equates to assigning all tied objects the best possible rank. The paragraph has been rewritten to clarify this point, see Comment 7.

9. Figure 7 – has missing axes labels and hence can't be interpreted.

We thank the reviewer for pointing out this omission. Scale has been added to the axes in Figure 7 and corresponding images in Supplementary Information.

10. How do the links given by standardized correlation score and IOKR vary? Is there a degree of overlap?

All of the scoring functions considered in this paper assign a score to each of the potential links between a GCF and an MF in the data set. Therefore, the overlap between the output of any two scoring functions is always going to be the complete set of potential links, because they are assigning scores to the same set. The question of how well the scoring functions agree is therefore rather a question of how well the resulting rankings correlate to one another.

While we do not evaluate the correlation between the different scoring functions, we do demonstrate that they are complementary, i.e. some verified links that are assigned a low score using IOKR, have a high score using the standardised strain correlation score, and vice versa, which is in our view a much more important property.

The complementarity between the two scoring functions is discussed extensively in Section 3.4.

l.254 (l.233): Added text to emphasise that both scoring functions are operating on the same set, so the overlap between the links is complete:

"Since both approaches work by considering the set of all possible GCF-MF combinations in the data set and assigning scores to each one, we can consider the scores for the links as assigning to each link a point in R2, given by (\sigma_corr, \sigma_IOKR)."

11. Table 4 - Since there are so many links above the validated links, what does it mean? Based on the claims in Results section 1, I would imagine that validated link from the combination of two scores (I_1/2) to have the highest rank (but do not observe it in Table 4). Shouldn't the validated links have a higher rank compared to the others? This should be discussed.

Being able to rely exclusively on automated ranking of potential links to find the correct ones is still a considerable way off. Validation of BGC-spectrum links relies on a combination of automated scoring, process of elimination, expert knowledge and trial and error. The fact that using the scoring functions, there are potential links that are higher ranked than the validated examples reflects this. Furthermore, as neither Molecular Networking nor BGC clustering into GCFs are completely solved problems, some of these links may actually be valid, although it is by no means certain.

While the I_1/2 scoring does not consistently place the validated links at the top of the ranking, it still outperforms the other scoring functions in the majority of cases, as indicated by the bold entries.

The product ordering, shown in the last column of Table 4, does generally assign the validated links better ranking than the other scoring functions. However, it has the major drawback of rendering a very large number of potential GCF-MF pairs incomparable, meaning that they can't be ranked amongst themselves.

As the ranking of links is displayed in Table 4, for a validated link, low numbers in the ranking are good, since they mean that a small number of links are considered more likely than the validated link.

The wording in the manuscript has been changed to avoid use of 'high rank', because of ambiguity - high rank could be seen either as referring to a high number (bad), or a low number (high on the ordered list of ranks, good).

l.596 (l.487): Added to paragraph to clarify that while I_1/2 does not consistently rank the validated link as the best one, it generally outperforms the individual scoring functions.

"While none of the scoring functions evaluated in Table 4 consistently ranks the validated links at the top of the set of potential links for the BGC in question, combinations of both scores, in particular I_1/2, tend to assign better rank to the validated links than the individual scoring functions."

Added to Table 4 caption that low numbers are good:

"Lower numbers indicate better ranking, and the best rank (excluding the product order) for each validated link is indicated in bold."

12. Also, since BGC0000137 has multiple entries, which rank should be prioritised and used for further analyses? This is an important point to be discussed in the manuscript.

The presence of multiple validated links for a single BGC in the dataset is a point well worth discussing and we thank the reviewer for pointing out this omission.

In the case of MIBiG ID BGC0000137, this multitude of links comes about because of three factors: The number of molecular families assigned to the MIBiG ID, the number of annotated BGCs with this MIBiG ID, and the predicted product type of the BGC, in this case

PKS-NRP Hybrid, giving rise to up to three different GCFs (since the BGC can be classified as NRPS, PKS or hybrid).

Given a starting BGC with a hybrid product type, resulting in multiple potential GCFs, it is up to the researcher to decide which GCF they consider most likely. This can for instance be influenced by the predicted product type of the GCF and the MS2 spectrum, or by the scoring functions.

l.422 (l.340): Added an explanation of the existence of multiple validated links in the Crüsemann data set:

“When clustering BGCs, BiG-SCAPE clusters the BGCs separately by product type. BGCs belonging to the class “PKS-NRP Hybrid” are treated as belonging to three classes: PKS, NRPS and Hybrid, and are therefore clustered three times. After clustering, identical GCFs are removed, so if identical clusters appear in for instance PKS and NRPS, that cluster only appears once in the resulting data. The final outcome is that a hybrid BGC can belong to one, two or three GCFs.
For instance, considering the Crüsemann data set at the GCF-MF level gives 10 validated links for the MIBiG ID BGC0000137. That BGC is linked to two MFs in the data set, and two BGCs from the relevant strain show significant homology to the MIBiG BGC. Because both of the BGCs are assigned the product class “PKS-NRP Hybrid”, BiG-SCAPE considers them as potential members of a PKS cluster, NRPS cluster or a hybrid cluster. This yields two potential clusters for one of the BGCs and three for the other BGC, giving a total of five potential GCFs linked to the two MFs, and therefore ten individual GCF-MS2 links.”

To clarify things further, columns showing the IDs of the BGC, GCF and MF for each link were added to Table 4.

13. Since the authors are proposing a new method, I would suggest that the comparison must be done with BGCs from other tools like DeepBGCs as well.

While DeepBGC does represent a substantially different method of BGC detection from antiSMASH, we maintain that the NPLinker framework itself is completely independent of the BGC detection method.

Validation that the IOKR score is functioning as expected is done independently of BGC detection tools (such as antiSMASH) using the combined GNPS/MIBiG data set, results of which can be found in Section 3.2: Evaluating the IOKR scoring function using MIBiG.

Similar validation of IOKR and the standardised strain correlation scores on microbial data sets is done at the GCF level, where the BGC clustering tools are not yet compatible with DeepBGC output, precluding comparison.

Furthermore, as DeepBGC claims to outperform antiSMASH on novel BGCs in particular, and the only BGCs that can be ranked using IOKR at the moment are BGCs for which structural predictions can be made with a degree of confidence, the output of antiSMASH and deepBGC is highly unlikely to be significantly different for the BGCs which IOKR can at present process.

l.9: Added to intro to emphasise the more exploratory nature of DeepBGC:

“The most popular tool for BGC prediction in microbial genomes is antiSMASH [2], although several more exploratory tools exist ([4, 6]).”

l.312-236: Added Section 2.8: Test data, describing which parts of the analysis are dependent on antiSMASH, which are not, and why the difference between antiSMASH and DeepBGC is unlikely to be significant:

“While NPLinker can in principle accept BGCs and GCFs from any source as input, at present it is implemented and tested using the BiG-SCAPE workflow, in order to make use of strain correlation scoring. As DeepBGC is not yet incorporated into the BiG-SCAPE workflow, evaluation on microbial data sets is carried out using antiSMASH-predicted BGCs, both in the case of the standardised strain correlation, where the GCF information is required, but also in the case of IOKR, where only the BGC information is required. In practice, this is not likely to present much of a problem: as the main improvement of DeepBGC on antiSMASH lies in improved detection of novel BGCs, and to evaluate IOKR, we must restrict our analysis to BGCs for which reliable structural predictions exist - i.e. BGCs with strong homology to MIBiG BGCs - the difference between antiSMASH and DeepBGC is likely to be very slight for the BGCs in the test set.
Separate validation on IOKR, on a data set not dependent on antiSMASH, is carried out on a data set created by matching manually curated BGCs from MIBiG [31] to manually curated spectra from GNPS [22] using structural annotations from the databases.“

14. The link to the tool NPLinker is not provided in the main manuscript. Hence, this couldn't be used/tested.

We thank the reviewer for pointing this omission out.

l.296 (l.260): Added “NPLinker code and documentation can be found at https://github.com/sdrogers/nplinker.”

Minor
1. At a few places such as Lines 452, the notations are not consistent

We thank the reviewer for pointing this error out to us, we have changed \hat{\sigma} to \bar{\sigma}} on l.452 and in Table 4.

2. At several places these terms are used Established links, validated links, verified links and it becomes difficult to comprehend. I suggest authors use consistent terms or explain what each of these terms mean in the supplement.

We appreciate the suggestion and have changed ‘verified’ and ‘established’ links to ‘validated’ in all cases.

3. Small typos need to be fixed (e.g. Summary prodcut -> product)

We have gone over the manuscript and corrected various typos.

Reviewer #3:

Abstract: Single paragraph is recommended.

We thank for the suggestion and have removed the paragraph break

There are several typos in the manuscript, “prodcut, unknnown, concenrated, compuational, peptidogenomcs, severly, to produces, “ etc.

We have gone over the manuscript and corrected various typos.

There are some acronyms such as RiPP, but the open form is not given in the manuscript.

We thank the reviewer for pointing out this omission.

l.60 (l.59) Added open form: changed

“These tools are each designed to be used for only a single product type such as NRPs, PKs or RiPPs, respectively.”

to

“These tools are each designed to be used for only a single product type such as nonribosomal peptides (NRPs), polyketides (PKs) or ribosomally synthesized and post-translationally modified peptides (RiPPs), respectively.”

Table 2 is first mentioned in line 357, but without any details on its rows (what is total score etc). This information is provided much later, when Table 2 is mentioned again. Please either remove the first referral or move the related explanations to the place where Table 2 is first mentioned.

We are grateful to the reviewer for the suggestion.

l.447 (l.357): Added a sentence, changing the beginning of the paragraph from:

“Rows two and three of Table 2 show the proportion of validated links among the top scoring links for the raw and standardised correlation scores. ”

to

“Table 2 shows the proportion of validated links among all links in the microbial data sets, both for all the potential links (first row), and for the links scoring above the 90th percentile using the various scoring functions. In particular, rows two and three show the proportion of validated links among the top scoring links for the raw and standardised correlation scores”

Crüsemann figure (Figure 6) is repeated in the supplementary file section 10. In the related supplementary figures for other datasets, please use red, not green, for verified points. Green is difficult to see.

We appreciate the suggestion and have changed labels in Supplementary Information images from green to red.

Table 4: What do the bold numbers show? This should be added to table caption.

We thank the reviewer for pointing out this omission. The bold numbers show the best rank for each validated link.

We have added to the table caption: “The best rank for each validated link is indicated in bold.”
