# Supplementary material for: Ranking microbial metabolomic and genomic links in the NPLinker framework using complementary scoring functions
Source: PLoS Comput Biol. 2021 May 4;17(5):e1008920. doi: 10.1371/journal.pcbi.1008920 (PMC8130963; doi:10.1371/journal.pcbi.1008920)
Supplement: Attachment — Submitted filename: NPLinker-review-2.docx [file pcbi.1008920.s010.docx]

This manuscript by Eldjárn and collaborators describes efforts to create a new bioinformatic tool that more accurately connects biosynthetic gene clusters to specific compounds in a paired metabolomic sample analyzed by tandem MS. This new tool creates a single numeric measure of correlation using two complementary methods: a correlation metric for presence/absence of features, and an analysis of the predicted structural features from the two data sets using a molecular fingerprint strategy (using antiSMASH and MIBiG). This involves a fairly complex algorithm that employs an Input Output Kernel Regression approach. Overall, this seems a valuable new tool for the community, and with attention to the concerns/suggestions below, should be appropriate for publication.

1. Several of the figures are incompletely described in the figure legend such that the precise meaning is not clear. For example, there is no description specifically identifying what the “red dots” are depicting in Figures 6 and 7. The meaning of Figure 8 is also not very clear, and requires a better explanation, including its legend. The authors should go through all of their figures and check that they are fully annotated or described in the Figure legends.
2. All of the results in this paper are reported in the aggregate, and therefore, it is quite abstract and theoretical. It would be very useful to show an example or two where an actual BGC is correlated to a specific MS2 spectrum as well as the compound giving rise to these data. That is, it would help to demonstrate the utility of the tool through illustration with a specific example. For example, could the authors find a rare GCF with good BGC predicted structural insights and then use NPLinker to predict its MS/MS spectra pair. By making sense of the fragments using the predicted structure and the paired spectrum, one can partially/fully confirm the match without the need for isolation and NMR analysis (although a rigorous structure identification would be ideal).
3. Why is “ranking links” used in the title instead of “connecting” or “linking” BGC to MS/MS? Additionally, it is suggested to refine the wording to “connecting known BGCs to cryptic MS/MS spectra” to make it clear to the reader what the tool can do and not do, and how it can be applied. It’s important to point out to the reader that this tool is not useful for classifying non-homologous/unknown BGCs.
4. Part of the validation set from Paired Omics DB includes metagenome-assembled genomes (MAGs). MAGs tend to have two issues: fragmentation, splitting BGCs into multiple contigs, and; contamination, maybe adding extra BGCs to the MAG. Regarding fragmentation, suggest checking if the test BGCs are on the edge of contigs or if they are considered complete. For contamination, suggest checking for each BGC in a strain if the GC content deviates too much from the average. For more detailed inspection, tetranucleotide signature and closest relative taxa from the database are also options to confirm that the BGCs are not from contaminants. A Table for the 22 BGCs in the test set from Paired Omics Data Platform (PoDP) would be ideal, containing at least BGC type, completeness and GC deviation. This table could be part of the supporting information.
5. Why do the authors use BiG-SCAPE if BiG-SLICE scales are much better? It would be interesting to check how the algorithm behaves with BiG-SLICE vs BiG-SCAPE.
6. Why have the authors selected only three datasets out of 70 public datasets with over 100 validated links? The testing size of 22 BGCs is very small and may not represent many other microbes.
7. Perhaps it would be good to show that the tool outperforms some other straight forward ways to match predicted structures with MS/MS spectra; for example, matching via Tanimoto scores of predicted structures, one can collect predicted structures for the queried spectra using *in silico* tools and match those to predicted structures from tested BGCs. Alternatively, matching via direct presence/absence using Jaccard index, one could create a matrix of presence/absence for GCFs and a matrix for MFs and then make links between rows using Jaccard index. NPLinker should be able to generate more correct links than these other methods. Benchmarking NPLinker to other methods is an important recommended revision to this manuscript.
8. It is concerning that the accuracy seems very low (12 to 29% in Table 3?) and the authors should make a comment on it. An indication of accuracy should also be discussed or provided in the abstract and in the introduction. Table 3 should also have top-n accuracy for raw correlation, standardized correlation, standardized correlation plus IOKR, instead of only for IOKR.
9. GNPS has 38,102 spectra with annotated SMILES; why not use it all instead of only 4,138?
10. “Assuming that similar BGCs give rise to similar compounds, we used this list in turn to assign one or more molecular structures to BGCs, according to how many high-scoring matches are found in MIBiG (or none, if no match is found).” It’s a risk assumption because a single gene difference can cause a profound structural change or no change at all. The “linear” InChI keys are a good way to match MIBiG BGC to GNPS MS/MS. However, aligning the BGC labels with the MS/MS labels is suggested in order to double check that the links are correct.
11. Cite [Metaminer](https://www.sciencedirect.com/science/article/abs/pii/S2405471219303126) instead of RippQUEST.
12. Why is the standardized score for one of the family pairs in Fig. 2 A zero? If that’s the case, it requires more explanation.
13. On page 11, lines 332-339, please indicate if there was a similarity threshold for a “known cluster blast” match to be consider a true annotation?
14. What is the difference between validated vs verified links? If these are being used as synonyms, the authors should select one and only use one to avoid confusion. For instance, the use of these two terms as column headers in Table 4 is very confusing.
15. In Table 4, does the last column indicate that in none of the cases the correct response was the first link? Or is that the ranking of the validated link? Does the term “higher” refer to a ‘better score’ or a ‘bigger number’ (which would have opposite meanings and conclusions)?
16. Page 2, line 12 – spelling error ‘unknown’
17. P3, l90 – change “on” to “an”
18. P10, l288-290 – it’s not clear why these number do not total up? Is it that there are redundant spectra, and many redundant BGCs, such that there are many fewer “unique” spectra or BGCs compared to the number of pairs? This needs some further explanation.
19. P18, l533 – the ending sentence seems unnecessarily limiting. Three types of specific biological activity are noted, but there are of course many other pharmaceutical classes wherein new medications are needed. A more general statement seems more appropriate, such as “…. novel chemistry that is much needed in diverse areas of human medicine and agriculture”.
